# Excessive firing of dyskinesia-associated striatal direct pathway neurons is gated by dopamine and excitatory synaptic input

**DOI:** 10.1016/j.celrep.2024.114483

**Published:** 2024-07-17

**Authors:** Michael B. Ryan, Allison E. Girasole, Andrew J. Flores, Emily L. Twedell, Matthew M. McGregor, Rea Brakaj, Ronald F. Paletzki, Thomas S. Hnasko, Charles R. Gerfen, Alexandra B. Nelson

**Affiliations:** 1 Neuroscience Graduate Program, UCSF, San Francisco, CA 94158, USA; 2 Kavli Institute for Fundamental Neuroscience, UCSF, San Francisco, CA 94158, USA; 3 Weill Institute for Neurosciences, UCSF, San Francisco, CA 94158, USA; 4 Department of Neurosciences, UCSD, La Jolla, CA 92093, USA; 5 Veterans Affairs San Diego Healthcare System, San Diego, CA 92161, USA; 6 Department of Neurology, UCSF, San Francisco, CA 94158, USA; 7 Laboratory of Systems Neuroscience, National Institute of Mental Health, Bethesda, MD 20892, USA; 8 Aligning Science Across Parkinson’s (ASAP) Collaborative Research Network, Chevy Chase, MD 20815, USA; 9 These authors contributed equally; 10 Lead contact

## Abstract

The striatum integrates dopaminergic and glutamatergic inputs to select preferred versus alternative actions. However, the precise mechanisms underlying this process remain unclear. One way to study action selection is to understand how it breaks down in pathological states. Here, we explored the cellular and synaptic mechanisms of levodopa-induced dyskinesia (LID), a complication of Parkinson’s disease therapy characterized by involuntary movements. We used an activity-dependent tool (FosTRAP) in conjunction with a mouse model of LID to investigate functionally distinct subsets of striatal direct pathway medium spiny neurons (dMSNs). *In vivo*, levodopa differentially activates dyskinesia-associated (TRAPed) dMSNs compared to other dMSNs. We found this differential activation of TRAPed dMSNs is likely to be driven by higher dopamine receptor expression, dopamine-dependent excitability, and excitatory input from the motor cortex and thalamus. Together, these findings suggest how the intrinsic and synaptic properties of heterogeneous dMSN subpopulations integrate to support action selection.

## In brief

Ryan et al. explore the cellular and synaptic mechanisms of levodopa-induced dyskinesia, a complication of Parkinson’s disease treatment. Using an activity-dependent mouse line to capture dyskinesia-associated neurons, they find a subset of striatal direct pathway neurons with high levodopa-evoked activity and enhanced dopamine signaling and excitatory synaptic input.

## Graphical Abstract

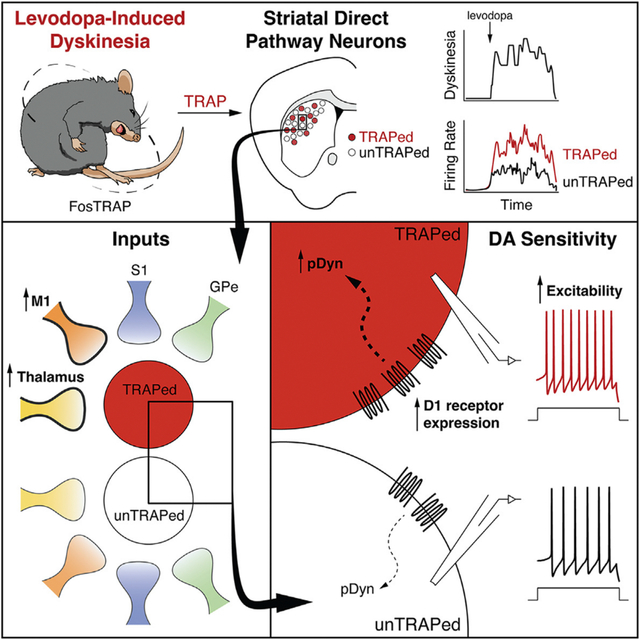

## INTRODUCTION

The striatum coordinates many behaviors, ranging from locomotion to reward-based decision-making. Within the striatum, two canonical classes of neurons, direct and indirect pathway medium spiny neurons (dMSNs and iMSNs, respectively) are thought to be central to executing such a diverse behavioral repertoire. Importantly, these two populations are distinguished by their projection targets and dopamine receptor expression.^1^ According to classical models of basal ganglia function, dMSNs promote movement, while iMSNs suppress it. Dopamine is hypothesized to bidirectionally modulate these striatal populations as a result of the segregated expression of “excitatory” D1-like dopamine receptors (G_s_-coupled) in dMSNs and “inhibitory” D2-like dopamine receptors (G_i_-coupled) in iMSNs.^1–3^ Accumulated evidence suggests balanced activity (co-activation) between these two populations supports normal action selection.^4–8^ However, an imbalance may contribute to impairments, resulting in movement or cognitive disorders.^9–11^ A key unanswered question in the field is how heterogeneous subpopulations *within* these two pathways contribute to action selection in health and disease.

One such disorder is levodopa-induced dyskinesia (LID), a complication of Parkinson’s disease (PD) in which prolonged treatment with the dopamine precursor levodopa results in abnormal involuntary movements. Work in nonhuman primate models of PD has identified abnormal striatal activity in LID,^12,13^ and cell-type-specific approaches in rodent models indicate LID is accompanied by increased dMSN and decreased iMSN activity, consistent with classical models.^14–16^ Furthermore, in keeping with the idea that direct and indirect pathways may contain functional subdivisions, we and others have observed considerable variability in how medium spiny neurons (MSNs) respond to levodopa and how their firing relates to dyskinesia severity *in vivo.*^12,15,17,18^ Previously, we identified a subpopulation of dMSNs *in vivo* with exceptionally high firing rates in response to levodopa, which in turn correlate with dyskinesia severity.^15^ In a parallel study, we used an activity-dependent transgenic mouse line (FosTRAP)^19^ to capture highly active neurons during a single episode of LID and found that re-activation of this subpopulation could drive dyskinesia in the absence of levodopa.^20^

Here, we used FosTRAP to explore the mechanisms that drive LID. We found that a subpopulation of dMSNs labeled by FosTRAP (TRAPed dMSNs) show exceptionally high levodopa-evoked firing, which correlates with dyskinesia severity on a moment-to-moment basis. To identify the intrinsic and synaptic changes that might underlie this aberrant firing pattern, we used Cre-dependent rabies tracing, optogenetics, *in vivo* and ex *vivo* physiology, and *in situ* hybridization. Using these approaches, we found that this subpopulation of TRAPed dMSNs has stronger excitatory synaptic input than neighboring dMSNs or iMSNs. TRAPed dMSNs also have higher expression of the D1 dopamine receptor and are more sensitive to D1 dopamine receptor activation than unTRAPed dMSNs. Together, these findings identify specific cellular mechanisms that underlie heterogeneous responses to levodopa, driving therapeutic effects as well as dyskinesias.

## RESULTS

**Figure 1.**
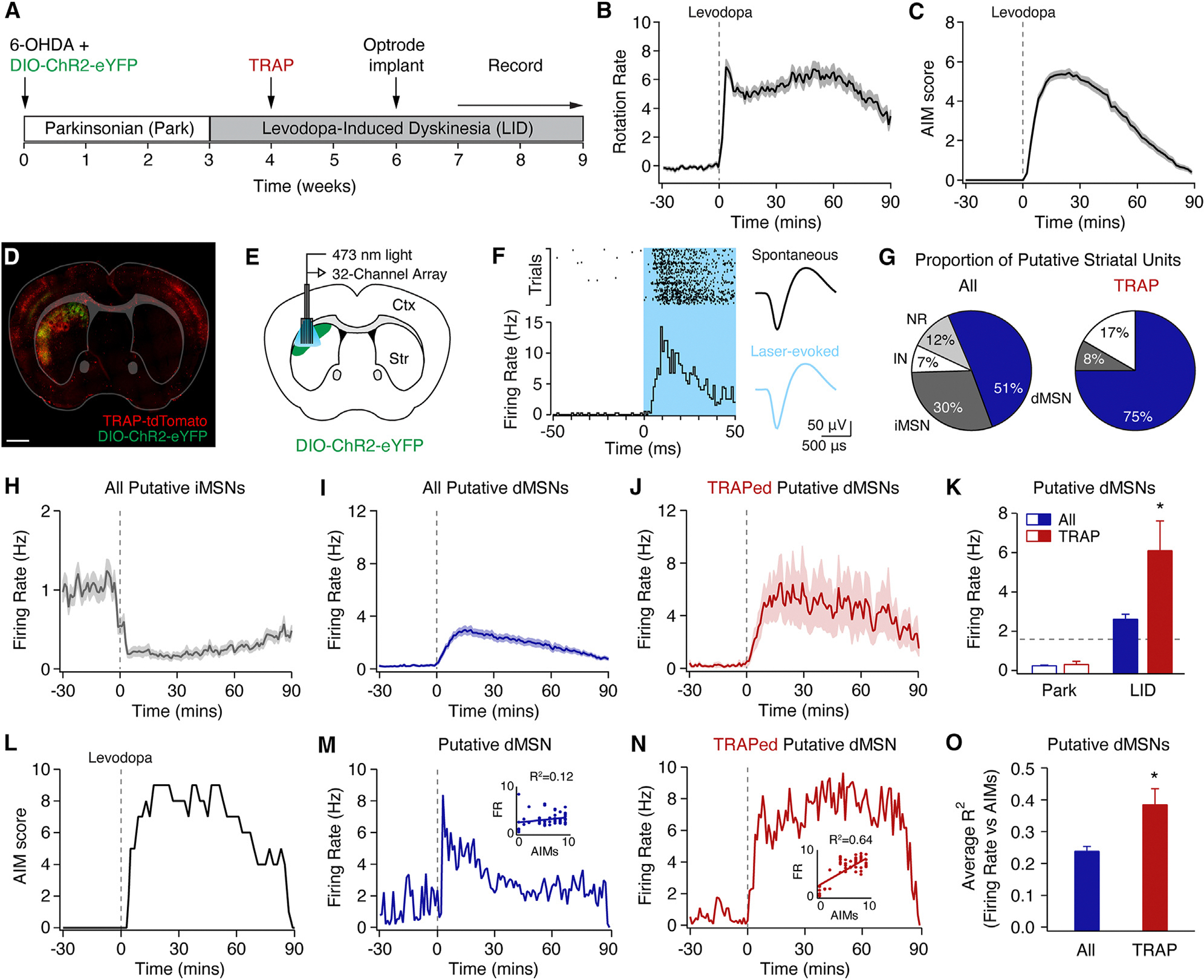
Optogenetically identified TRAPed striatal neurons show differential firing responses to levodopa *in vivo* TRAPed striatal single units were recorded in freely moving parkinsonian mice using an optogenetic labeling approach. (A) Experimental timeline. (B and C) Behavior in parkinsonian mice, aligned to levodopa injection at t = 0 (*N* = 20). (B) Rotation rate (contralesional-ipsilesional rotations per minute). (C) Dyskinesia (quantified by the Abnormal Involuntary Movement, AIM score). (D and E) DIO-ChR2-eYFP viral injection and optrode array in the dorsolateral striatum of a FosTRAP;Ai14 mouse. (D) Representative postmortem histology. Scale bar represents 1 mm. (E) Coronal schematic. (F) Representative optogenetically labeled TRAPed striatal unit. Left: peri-event raster (top) and histogram (bottom) showing spiking in response to laser (blue box). Right: average spontaneous (top) and laser-evoked (bottom) waveforms. (G) Proportion of all (left; *n* = 335, *N* = 20) and optically labeled TRAPed (right; *n* = 12, *N* = 9) striatal units, including putative interneurons (IN), direct pathway (dMSN), indirect pathway (iMSN), and no response (NR) striatal units. (H–J) Average firing rate of putative (H) iMSNs (*n* = 101, *N* = 20), (I) dMSNs (*n* = 170, *N* = 20), and (J) optically labeled TRAPed dMSNs (*n* = 9, *N* = 7), aligned to levodopa injection at t = 0. (K) Average firing rate of all putative dMSNs and optogenetically labeled TRAPed dMSNs in parkinsonian mice before (Park) and after levodopa administration (LID). Dotted line represents the firing rate of optogenetically labeled dMSNs from healthy controls (from Ryan et al.^15^). (L–N) Data from a single recording session, including (L) dyskinesia score, (M) firing rate of a putative dMSN, and (N) firing rate of an optogenetically labeled TRAPed putative dMSN. Insets: firing rate vs. dyskinesia score. (O) Average correlation (R^2^) of firing rate to dyskinesia for all putative dMSNs vs. TRAPed dMSNs. *n* = single units, *N* = mice. All data are presented as mean ± SEM. *p < 0.05, Wilcoxon rank-sum test. See also Figure S1.

A summary of all statistical comparisons can be found in Table S1. To investigate the physiological drivers of LID, we captured LID-associated striatal neurons using FosTRAP^CreER^ in a toxin-based mouse model of PD and LID (Figure 1A).^21^ The left medial forebrain bundle of FosTRAP;Ai14 (TRAP) mice was injected with the dopaminergic neurotoxin 6-hydroxydopamine (6-OHDA), leading to loss of midbrain dopamine neurons (Figure S1A), ipsilateral rotational bias, and reduced movement velocity (Figures S1B and S1C). Several weeks later, mice began daily levodopa treatment, resulting in contralateral rotational bias, increased movement velocity, and robust LID (Figures 1B, 1C, S1C, and S1D). LID-associated neurons were captured (“TRAPed”) 1 week into the treatment course with coadministration of levodopa and 4-OH tamoxifen, driving Cre-dependent expression of tdTomato (Figure 1D).^20^ TRAPed LID-associated neurons were a small subset of striatal neurons, with the highest density (~10%) in the dorsolateral striatum (DLS; Figure S1E), consistent with patterns seen using cFos immunohistochemistry.^20,22,23^ As previously reported, neurons TRAPed during LID are also highly enriched for dMSNs (~75%; Figures S1F and S1G), compared to other striatal subtypes.^20^

### TRAPed direct pathway neurons differentially respond to levodopa *in vivo*

Levodopa changes both firing rate and pattern in MSNs in animal models of PD.^12,13,15^ To compare the firing of TRAPed cells to other striatal neurons, we performed optogenetically labeled single-unit recordings in the DLS of freely moving parkinsonian mice (Figure 1E). TRAPed neurons were optically identified by their short-latency light-evoked responses (Figure 1F).^24^ Of a total of 335 single units recorded in 20 FosTRAP^CreER^ mice, we identified 12 optically identified TRAPed neurons that met our inclusion criteria. In line with previous findings, we observed a variety of responses to levodopa across striatal neurons, including decreased firing (Figures S1H–S1I), increased firing (Figures S1J–S1K), and no significant change (Figure S1L). Based on previous optically labeled recordings in a similar model, we classified these units as putative iMSNs, dMSNs, or NR (no response) units (Figures 1G–1I).^15^ We also identified putative interneurons based on spike waveform (Figures S1M and S1N).^25,26^ We found TRAPed neurons were highly enriched for dMSNs compared to the overall population (Figure 1G), as predicted by histological characterization (Figures S1F and S1G).^20^ In the parkinsonian state, TRAPed neurons and other dMSNs had comparably low firing rates, but in response to levodopa, TRAPed neurons achieved significantly higher firing rates (Figures 1I–1K and S1O).

We next examined the firing dynamics of TRAPed dMSNs. Consistent with previous work, we found the firing of some, but not all, dMSNs correlates with dyskinesia severity (Figures 1L–1N).^15^ Given the causal link between activation of TRAPed dMSNs and dyskinesia,^20^ we hypothesized their firing would be more tightly correlated with dyskinesia severity. Indeed, we found that compared to all dMSNs, the firing of TRAPed dMSNs was more correlated with dyskinesia (Figures 1O and S1P). These findings indicate that the firing of TRAPed dMSNs is not only more sensitive to levodopa but correlates with dyskinesia on a moment-to-moment basis. Together, these results indicate TRAPed neurons represent a distinct subpopulation of striatal MSNs in LID.

### Monosynaptic rabies tracing reveals reduced number of excitatory inputs onto TRAPed direct pathway neurons in LID

**Figure 2.**
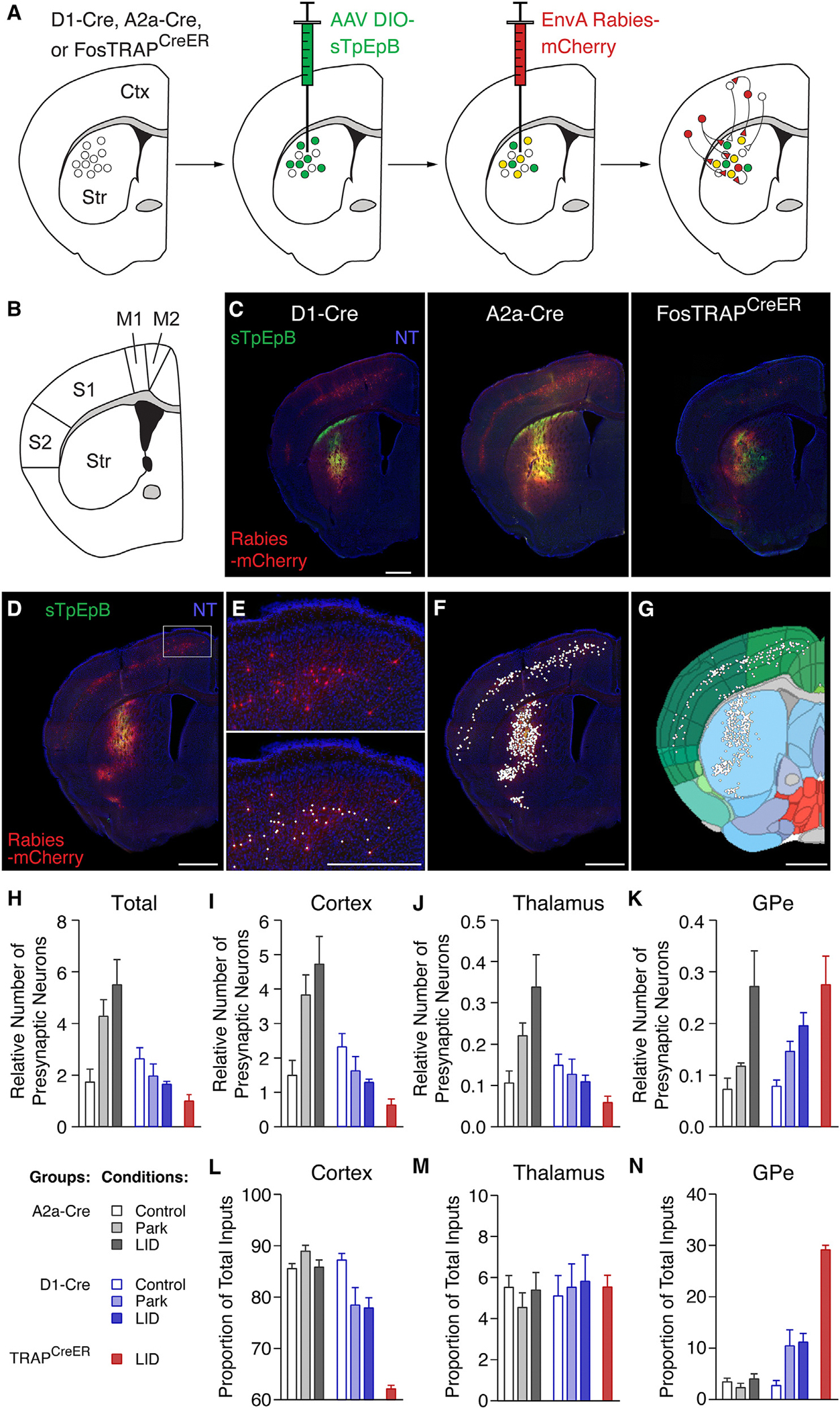
Monosynaptic rabies tracing of striatal inputs in healthy, parkinsonian, and levodopa-treated mice A dual viral, Cre-dependent strategy was used to label monosynaptic inputs onto direct pathway, indirect pathway, and TRAPed striatal neurons. (A) Schematic of the experimental approach in D1-Cre, A2a-Cre, and FosTRAP^CreER^ mice. (B and C) Coronal schematic (B) and low-magnification histological sections (C) showing helper virus expressing “starter” neurons (sTpEpB, green) and rabies-labeled neurons (Rabies-mCherry, red) in D1-Cre (left), A2a-Cre (middle), and FosTRAP^CreER^ (right) mice. NT = NeuroTrace stain for visualization. (D–G) Quantification of labeled presynaptic cell bodies. (D) Low-magnification image of coronal section showing helper (green) and rabies (red) viral injection sites. (E) High magnification of the box in (D), with overlaid points denoting rabies-positive presynaptic cell bodies (bottom). (F and G) Same section as in (D) with overlaid cell detection for (F) the histological section and (G) the projection into the Allen Brain Atlas. (H–K) Relative number of presynaptic neurons, calculated as the number of extra-striatal rabies-labeled neurons divided by the number of co-infected (rabies/helper virus labeled) striatal neurons for (H) all extra-striatal brain regions, (I) cortex, (J) thalamus, and (K) external globus pallidus. (L–N) Relative proportion of total inputs, calculated as the number of extra-striatal rabies-labeled neurons divided by the total number of extra-striatal labeled neurons brain-wide for (L) cortex, (M) thalamus, and (N) external globus pallidus. A2a: control, *N* = 6, Park, *N* = 4, LID, *N* = 3; D1: control, *N* = 9, Park, *N* = 10, LID, *N* = 6; TRAP: LID, *N* = 8. Data are presented as mean ± SEM. *N* = animals. Scale bars represent 1 mm. See also Figures S2, S3, S4, and S5A–S5D.

Striatal MSNs are highly dependent on excitatory synaptic input to drive spiking.^27^ To determine whether the number or distribution of synaptic inputs to TRAPed dMSNs might contribute to their *in vivo* firing dynamics and more broadly to LID, we performed Cre-dependent rabies tracing^28^ from the DLS (Figure 2A). We used D1-Cre, A2a-Cre, and FosTRAP^CreER^ mice to enable comparisons between inputs to dMSNs, iMSNs, and TRAPed striatal neurons, respectively (Figures 2B and 2C). Striatal “starter” cells and presynaptic rabies-labeled cell bodies were detected, mapped onto the Allen Brain Atlas, and quantified by brain region (Figures 2D–2G, S2, and 3A–D).^29^ To quantify changes in the number of presynaptic neurons, we first calculated the relative number of presynaptic neurons to co-infected striatal starter cells (Figure S3D) across healthy, parkinsonian, and LID conditions (referred to hereafter as “number,” Figures 2H–2K). Additionally, to quantify changes in the overall distribution of presynaptic neurons across the entire brain, we also calculated the relative proportion of presynaptic neurons in one brain region versus the total number of extra-striatal presynaptic neurons brain-wide (referred to hereafter as “proportion,” Figures 2L–2N and S3E). In the healthy condition, monosynaptic inputs onto dMSNs and iMSNs showed a similar number of presynaptic neurons (Figure 2H), the majority of which derive from the ipsilateral cortex, thalamus, and external segment of the globus pallidus (GPe) (Figures 2I–2N and S3E), as has been previously reported.^30,31^

In parkinsonian mice, we observed opposing changes in synaptic inputs onto iMSNs and dMSNs. Compared to healthy mice, iMSNs in parkinsonian mice showed an increase in the number of cortical presynaptic neurons, with no significant change in the number of thalamic or GPe presynaptic neurons (Figures 2I–2K), leading to an overall *increase* in the proportion of cortical inputs to iMSNs (Figure 2L). In contrast, dMSNs showed no change in the number of cortical and thalamic presynaptic neurons but an increase in the number of presynaptic GPe neurons (Figures 2I–2K). This increase in GPe inputs led to a *decrease* in the overall proportion of cortical inputs to dMSNs in parkinsonian mice (Figure 2L). Levodopa-treated parkinsonian animals showed similar overall patterns of monosynaptic inputs to those seen in untreated parkinsonian mice. In iMSNs, there was no significant change in the number of cortical or thalamic presynaptic neurons (Figures 2I and 2J). However, there was an increase in the number of presynaptic GPe neurons compared to untreated parkinsonian mice (Figure 2K). In dMSNs, we observed no significant changes in the pattern of monosynaptic inputs in levodopa-treated, compared to untreated, parkinsonian animals (Figures 2I–2K). Together, these results suggest that, while dopamine depletion leads to opposing changes in the inputs onto dMSNs and iMSNs, chronic levodopa administration does not markedly change the distribution of monosynaptic inputs onto these canonical MSN subclasses in parkinsonian mice.

To investigate the possible synaptic drivers of aberrant activity of TRAPed neurons during LID, we next compared the monosynaptic inputs onto TRAPed neurons versus all dMSNs, focusing on levodopa-treated parkinsonian mice. We hypothesized that TRAPed neurons might receive inputs from a larger number of excitatory cortical and thalamic neurons. While the total number of presynaptic neurons brain-wide was similar between TRAPed neurons and all dMSNs (Figure 2H), contrary to our hypothesis, TRAPed neurons had a smaller number of inputs from excitatory sources (thalamus and cortex) than did dMSNs more broadly (Figures 2I and 2J). This reduction in the number of presynaptic cortical neurons was observed across most motor and somatosensory cortices, leading to a striking reduction in the proportion of cortical inputs (Figure 2L and S3E). TRAPed neurons also showed a similar number of presynaptic GPe neurons compared to dMSNs during LID (Figure 2K). The majority of GPe neurons projecting to TRAPed striatal neurons were Npas1 positive (~50%) with only a small fraction (~1%) being parvalbumin (PV) positive (Figures S5A–S5D), in line with previous reports characterizing striatal-projecting arkypallidal neurons.^32–36^ Taken together, these findings suggest that chronic changes in dopamine produce opposing changes in synaptic inputs onto iMSNs and dMSNs, with a marked loss in the number of corticostriatal neurons synapsing onto TRAPed neurons (and dMSNs) in LID. Contrary to our initial hypothesis, this observation is unlikely to explain the high levodopa-evoked firing of TRAPed neurons *in vivo*.

### Synaptic physiology shows enhanced motor cortical and thalamic excitatory input onto TRAPed direct pathway neurons in LID

**Figure 3.**
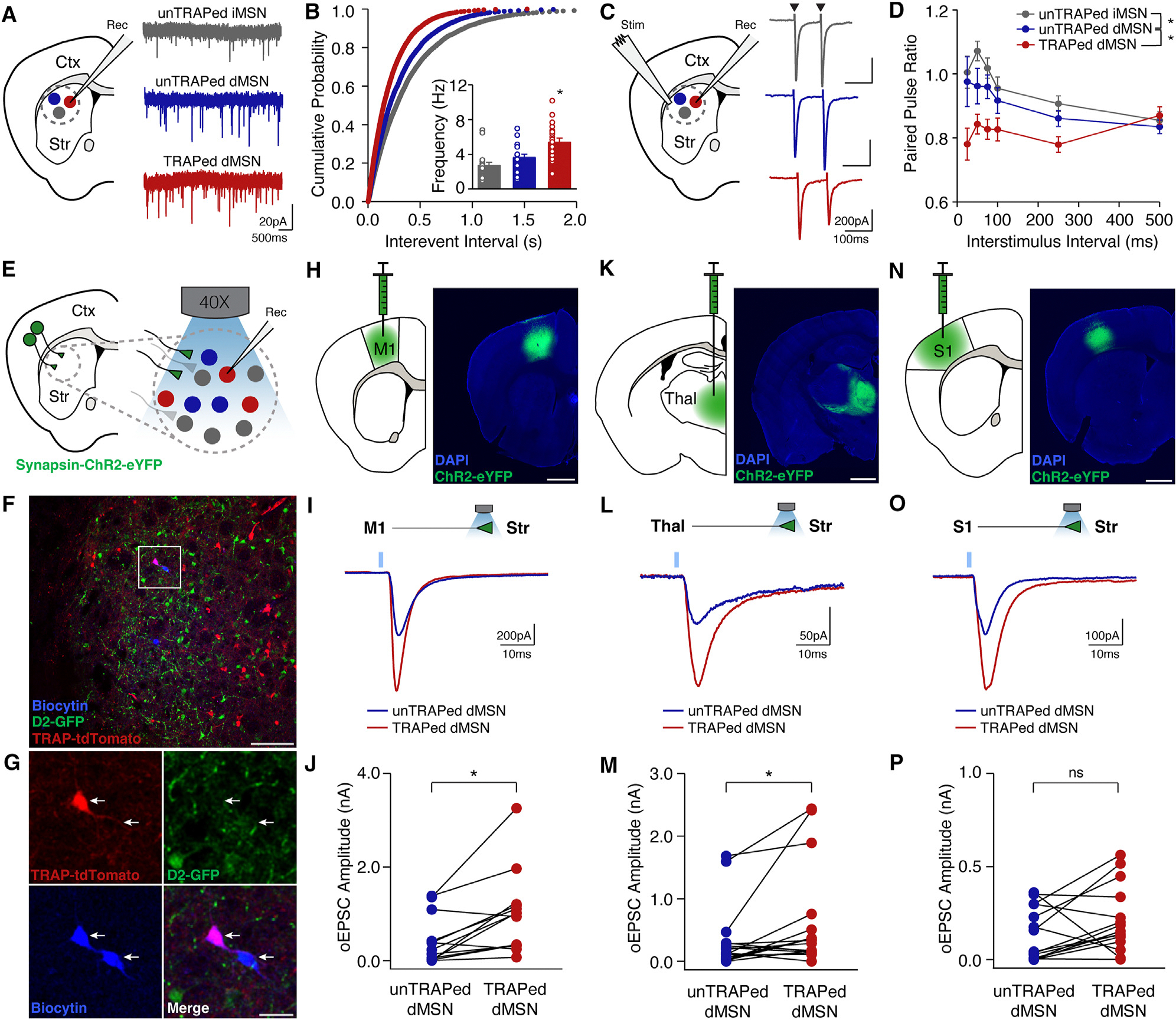
Increased presynaptic excitatory transmission onto TRAPed dMSNs Excitatory inputs to striatal dMSNs were compared in *ex vivo* brain slices from the dorsolateral striatum of FosTRAP;Ai14;D2-GFP mice. (A–D) (A) Coronal schematic (left) and representative current traces (right) from unTRAPed iMSNs, unTRAPed dMSNs, and TRAPed dMSNs in the presence of picrotoxin and tetrodotoxin to isolate miniature excitatory postsynaptic currents (mEPSCs). (B) Cumulative probability distribution of mEPSC frequencies. Inset: average mEPSC frequencies (unTRAPed iMSNs: *n* = 21, *N* = 8; unTRAPed dMSNs: *n* = 20, *N* = 7; TRAPed dMSNs: *n* = 20, *N* = 8). (C) Coronal schematic (left) and representative traces (right) from unTRAPed iMSNs, unTRAPed dMSNs, and TRAPed dMSNs in response to local electrical stimulation (arrowheads) in the presence of picrotoxin to isolate evoked EPSCs. (D) Quantification of the paired pulse ratio in unTRAPed iMSNs (*n* = 17, *N* = 8), unTRAPed dMSNs (*n* = 18, *N* = 9), and TRAPed dMSNs (*n* = 22, *N* = 9). (E–P) The strength of major excitatory inputs was compared across unTRAPed and TRAPed dMSNs using an optogenetic approach, *n* = pairs, *N* = mice. (E) Schematic of recording configuration. (F) Low-magnification image of dorsolateral striatum, showing TRAPed neurons (red), D2R-expressing neurons (green), and a pair of biocytin-filled neurons (blue). Scale bar represents 100 μm. (G) High magnification of section in (F), showing a pair of neighboring, sequentially recorded TRAPed and unTRAPed dMSNs (arrows). Scale bar represents 25 μm. (H–P) Optical activation of inputs from primary motor cortex (H–J, M1, *n* = 19, *N* = 4), thalamus (K–M, Thal, *n* = 15, *N* = 5), and primary somatosensory cortex (N–P, S1, *n* = 13, *N* = 7). (H, K, and N) Coronal schematics (left) and postmortem histology (right) showing viral expression of ChR2-eYFP. Scale bar represents 1 mm. (I, L, and O) Representative examples of optically evoked EPSCs (oEPSCs) for an unTRAPed and TRAPed dMSN (bottom). (J, M, and P) Average oEPSC amplitude at 4 mW for unTRAPed and TRAPed dMSNs. *p < 0.05, Wilcoxon rank-sum test. *n* = cells, *N* = mice. Data are presented as mean ± SEM. See also Figures S4 and S5E–S5H.

Though rabies tracing showed structural differences in excitatory synaptic input onto TRAPed dMSNs, this approach has several biases and may fail to capture synaptic *function*. To directly compare the strength of excitatory synapses onto neighboring TRAPed dMSNs, unTRAPed dMSNs, and unTRAPed iMSNs, we used *ex vivo* slice recordings in FosTRAP;Ai14;D2-GFP mice. Based on the tight correlation of TRAPed dMSN spiking to dyskinesia *in vivo*, we hypothesized that excitatory input would be increased onto TRAPed neurons. We tested this hypothesis with several electrophysiological measures that reflect pre- and postsynaptic function. To assess presynaptic changes, we first measured miniature excitatory postsynaptic currents (mEPSCs), which were increased in frequency, but not amplitude, in TRAPed dMSNs (Figures 3A, 3B, S4A, and S4B), consistent with a possible increase in the number of presynaptic terminals or the probability of vesicle release. To assess for postsynaptic changes, we next measured electrically evoked excitatory postsynaptic currents (EPSCs). We found no differences in the AMPA:NMDA ratio (Figures S4C and D). However, in the same recordings, we found a marked decrease in the paired pulse ratio (PPR) in TRAPed dMSNs compared to unTRAPed dMSNs or iMSNs (Figures 3C and 3D). Together, these results suggest a higher probability of release at excitatory synapses onto TRAPed dMSNs compared to other MSNs.

While these findings suggest greater strength of excitatory input onto TRAPed dMSNs, they do not identify the specific source. We next assessed the strength of several key inputs to the DLS using an optical approach (Figures 3E–3G). We injected hSyn-ChR2 into primary motor cortex (M1), somatosensory cortex (S1), thalamus (Thal), or GPe (Figures 3H–3K, 3N, and S5E) to measure optically evoked excitatory and inhibitory postsynaptic currents (oEPSCs and oIPSCs, respectively). Using sequential paired recordings in the DLS, we found oEPSCs from M1 and Thal were larger onto TRAPed versus neighboring unTRAPed dMSNs (Figures 3H–3M and S4E–S4H). However, S1 oEPSCs and GPe oIPSCs were not statistically different between groups (Figures 3N–3P; S4I, S4J, and S5F–S5H). These results indicate TRAPed dMSNs receive greater excitatory synaptic input from motor cortical and thalamic brain regions.

### TRAPed direct pathway neurons are more sensitive to dopamine signaling

**Figure 4.**
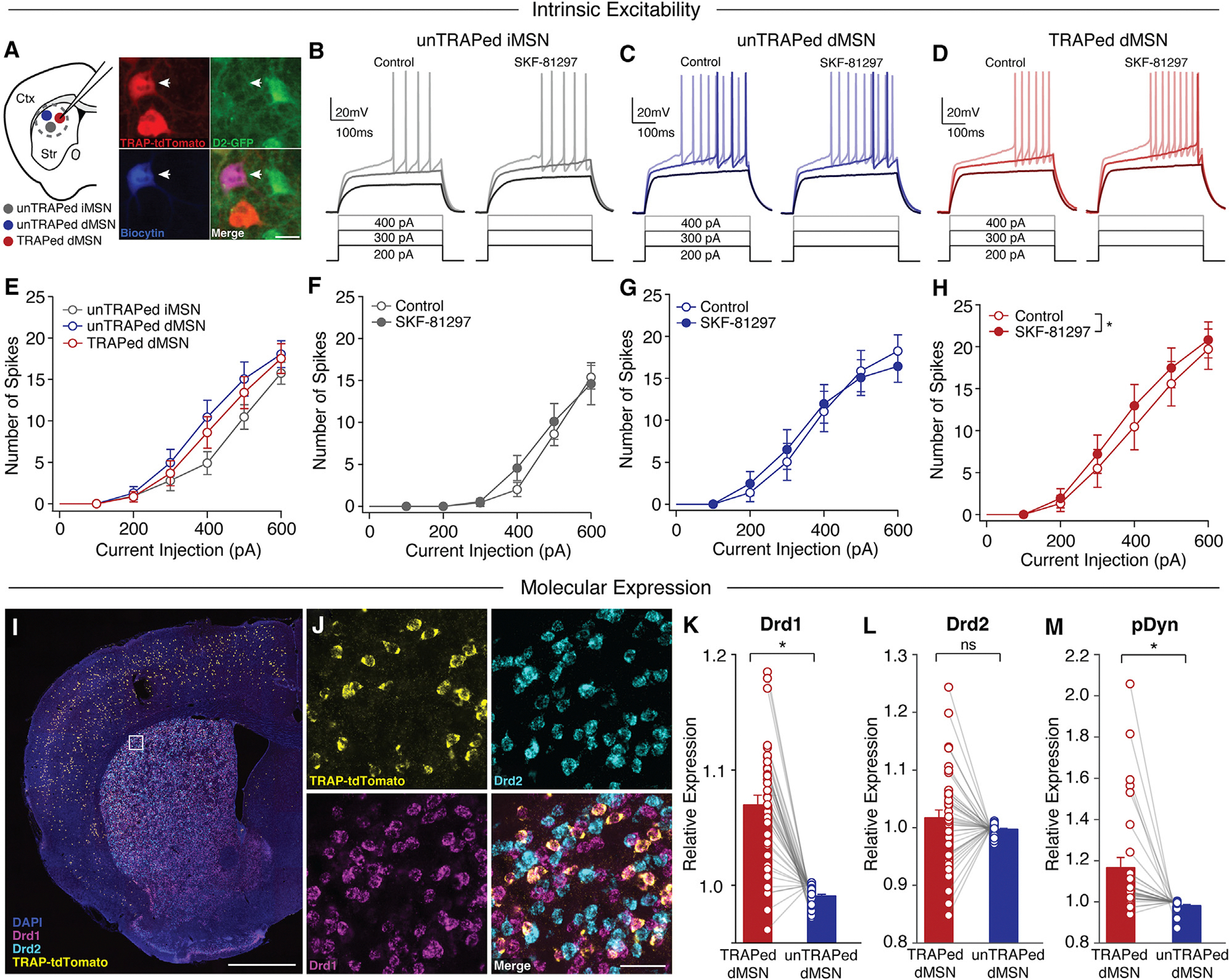
Activation of dopamine D1 receptors enhances the excitability of TRAPed, but not unTRAPed, dMSNs (A–H) Striatal neurons were targeted for *ex vivo* whole-cell recordings in coronal brain slices from parkinsonian and levodopa-treated FosTRAP;Ai14;D2-GFP mice. (A) Left: cartoon showing recordings of unTRAPed iMSNs (gray), unTRAPed dMSNs (blue), and TRAPed dMSNs (red) in the dorsolateral striatum. Right: histological image showing a biocytin-filled TRAPed dMSN targeted for recording (arrow). Tissue shows expression of FosTRAP;Ai14 (tdTomato, red), D2R (GFP, green), and biocytin (blue). Scale bar represents 20 μm. (B–D) Representative voltage responses to current injections before (left) and 10–15 min after bath application of the D1R-agonist, SKF-81297 (right) for an (B) unTRAPed iMSN, (C) unTRAPed dMSN, and (D) TRAPed dMSN. (E) Average current-response curves for unTRAPed iMSNs (gray, *n* = 17, *N* = 11), unTRAPed dMSNs (blue, *n* = 17, *N* = 13), and TRAPed dMSNs (red, *n* = 22, *N* = 14). *N* = cells, *N* = mice. *p < 0.05, rmANOVA. (F–H) Current-response curves before (control) and 10–15 min after SKF-81297 for (F) unTRAPed iMSNs (*n* = 9, *N* = 6), (G) unTRAPed dMSNs (*n* = 11, *N* = 9), and (H) TRAPed dMSNs (*n* = 14, *N* = 10). *n* = cells, *N* = mice (I–M) Fluorescent *in situ* hybridization to quantify D1 dopamine receptor (Drd1a), D2 dopamine receptor (Drd2a), and prodynorphin (pDyn) mRNA in levodopa-treated FosTRAP;Ai14 mice. (I) Low magnification of coronal section labeled for DAPI, D1R (Drd1), D2R (Drd2), and TRAP (tdTomato) mRNA. Scale bar represents 1 mm. (J) High magnification of inset shown in (I). Scale bar represents 50 μm. (K–M) Expression of molecular markers for TRAPed and unTRAPed dMSNs, normalized by the average expression of all dMSNs. (K) Quantification of relative expression of D1R mRNA in TRAPed and unTRAPed dMSNs (*n* = 39, *N* = 5). (L) Quantification of relative expression of D2R mRNA in TRAPed and unTRAPed dMSNs (*n* = 39; *N* = 5). (M) Quantification of relative expression of pDyn mRNA in TRAPed and unTRAPed dMSNs (*n* = 31; *N* = 4). *n* = slices, *N* = mice. Data are presented as mean ± SEM. See also Tables S2 and S3. See also Figure S6. *p < 0.05, Wilcoxon signed rank test.

Our *in vivo* findings suggested TRAPed dMSNs are a subpopulation with distinct physiological responses to levodopa. These responses might be mediated by differences in intrinsic properties or sensitivity to dopamine signaling. To address these possibilities, we again made *ex vivo* whole-cell patch-clamp recordings from identified DLS TRAPed dMSNs, unTRAPed dMSNs, and iMSNs (Figure 4A). In current-clamp recordings, we found that basic properties did not differ between the three cell types (Figures 4B–4E and Table S2). However, we hypothesized that D1R activation would increase the excitability of TRAPed dMSNs based on previous studies showing similar effects in healthy animals.^37–39^ Application of the D1R agonist SKF81297 increased the excitability of TRAPed dMSNs, while unTRAPed iMSNs and dMSNs showed no significant change (Figures 4F–4H). This increased excitability in TRAPed dMSNs included both a significant decrease in rheobase (minimum current needed to elicit spiking; Table S3) and increased firing rate in response to current injection (Figure 4H) following bath application of SKF-81297, suggesting TRAPed dMSNs are more sensitive to dopamine receptor activation.

Greater dopamine-dependent enhancement of TRAPed dMSN excitability may be driven by differential expression of D1 dopamine receptors and/or amplification of downstream signaling. To test these possibilities, we first compared dopamine receptor expression between TRAPed and unTRAPed dMSNs in the same animals by performing fluorescent *in situ* hybridization for mRNA encoding D1 (Drd1) and D2 (Drd2) dopamine receptors in parkinsonian FosTRAP;Ai14 mice chronically treated with levodopa (Figures 4I and 4J). Indeed, we found that TRAPed dMSNs showed a highly significant enrichment for D1, but not D2, dopamine receptors compared to unTRAPed dMSNs (Figures 4K and 4L). To determine if TRAPed dMSNs also showed greater signaling downstream of the D1 dopamine receptor, we performed fluorescent *in situ* hybridization for prodynorphin (pDyn), a transcription factor whose expression is upregulated following D1R activation and correlated with dyskinesia severity in parkinsonian animals.^40,41^ We found that TRAPed dMSNs also exhibited significantly higher pDyn levels than unTRAPed dMSNs within the same region (Figures 4M, S6A, and S6B). Furthermore, increased D1R and pDyn mRNA expression in TRAPed dMSNs was most prominent in the DLS compared to the DMS and VLS (Figures S6C–S6E). Together, these findings suggest that greater dopamine sensitivity, combined with a selective enhancement of excitatory inputs, might underlie the excessive levodopa-evoked firing of TRAPed dMSNs *in vivo*.

## DISCUSSION

To explore the mechanisms and consequences of heterogeneity within one of the canonical striatal pathways, the direct pathway, we used FosTRAP^CreER^ in a mouse model of LID. Previous work has described striatal subpopulations, based on molecular identity, anatomical location, inputs or outputs,^42–48^ but connecting these features to functional roles *in vivo* has been more challenging. Using FosTRAP^CreER^ to label neurons whose activation is known to cause dyskinesia, we were able to directly test whether *in vivo* or *ex vivo* changes associated with LID are differentially expressed between dMSNs.

We found that *in vivo*, optically identified TRAPed neurons were enriched for a subpopulation of DLS dMSNs previously identified by their firing and functional features.^15^ The transition from low to exceptionally high levodopa-evoked firing rates in these neurons may also explain the large levodopa-activated dMSN ensembles seen in a recent calcium imaging study in parkinsonian mice.^49^ Though our recordings were performed in a mouse model of PD with pharmacological manipulations, our findings fit into a broader literature identifying very diverse activity patterns in both healthy and disease model mice. We found that TRAPed neurons differ from other dMSNs by virtue of their firing rate in response to levodopa and their pattern of firing (i.e., correlation with dyskinetic behavior). In healthy animals, using single-unit recordings or miniscope recordings using fluorescent calcium indicators, other groups have found that striatal neurons have diverse and distinct tunings, including representing elapsed time,^50^ movement speed^51^ in trained tasks, and may indeed switch these tunings across tasks.^52^ In parkinsonian mice, other groups have identified interesting patterns of co-activation across neurons which likely relate to behavioral state.^18,49^ While TRAPed dMSNs represent a subset of all dMSNs, our findings suggest they both correlate with and cause LID in parkinsonian mice. However, a key question remains—what are the underlying mechanisms that produce this distinct phenotype? Two major possibilities to explain this phenomenon are intrinsic excitability and/or synaptic input.

The *in vivo* properties of TRAPed dMSNs could be explained by increased intrinsic excitability, making them more likely to spike to a given synaptic input. Previous work has found increases in dMSN excitability in chronically parkinsonian mice and only partial normalization of excitability in levodopa-treated animals.^53^ Additionally, a recent study found heterogeneity in intrinsic excitability between MSNs in a mouse model of LID.^17^ However, we found that basal excitability was quite similar between TRAPed and unTRAPed dMSNs. This matches well with our observation that *in vivo*, dMSNs show uniformly low firing rates in the parkinsonian condition—only upon levodopa administration are the differences between dMSNs apparent. Indeed, we found that compared to unTRAPed dMSNs, TRAPed dMSNs showed a modest increase in excitability in response to dopamine receptor activation. This differential response might be mediated by increased D1 receptors and/or their downstream signaling, regulating excitability in TRAPed dMSNs.^23,38,54,55^ However, there is conflicting evidence regarding increased D1 dopamine receptor expression in LID.^55–60^ While we did not compare D1 receptor expression across states, we used *in situ* hybridization to compare across cell types within the same animals, finding that TRAPed dMSNs show higher D1R mRNA expression compared to their unTRAPed dMSN neighbors. In the striatum, chronic D1R stimulation in both healthy and parkinsonian animals also leads to robust upregulation of dynorphin and prodynorphin (pDyn) levels.^1,61,62^ Increased pDyn levels are also highly correlated with dyskinesia severity.^40,61,63^ Accordingly, greater D1R expression in TRAPed neurons might lead to greater downstream signaling. In line with this hypothesis, in dyskinetic mice, we found higher pDyn mRNA levels in TRAPed dMSNs compared to unTRAPed dMSNs. Given these findings, LID-dependent changes in dopamine receptors, and their associated downstream signaling, are unlikely to be homogeneous across dMSNs; this phenomenon may lead to conflicting results or the lack of detected changes, depending on the sampling of different cell types in the overall population. Indeed, recent work suggests enhanced signaling downstream of the D1R in LID is regulated in a subregion-specific manner, with the largest effect in the DLS.^64^ As levodopa treatment would be expected to increase local dopamine signaling, the resulting increase in excitability might amplify the responses of TRAPed dMSNs to their synaptic inputs *in vivo* compared to their neighboring unTRAPed dMSNs.

In addition, the excitatory synaptic inputs onto TRAPed dMSNs may be enhanced, particularly from sensorimotor areas that are likely to drive voluntary (and involuntary) movements. Previous studies have used slice electrophysiology to examine excitatory synaptic inputs onto striatal neurons in rodent models of PD and LID, finding on the one hand an increase in synaptic strength onto MSNs overall^65^ and on the other a decrease in synaptic input onto dMSNs specifically.^53^ Here, we used anatomical (monosynaptic rabies tracing) and physiological assays (slice electrophysiology) to investigate alterations in synaptic input onto TRAPed MSNs. Rabies tracing revealed opposing changes in the presynaptic inputs onto dMSNs and iMSNs between the healthy and parkinsonian conditions, which were not restored with chronic levodopa administration. However, this approach did not find increases in the relative number of excitatory presynaptic neurons targeting dMSNs or TRAPed dMSNs in LID. In fact, the overall *reduction* in presynaptically labeled neurons may relate to the reductions in spine density seen on dMSNs in a mouse model of LID.^53^ However, slice electrophysiology indicated these inputs were much stronger in TRAPed dMSNs versus their unTRAPed dMSN neighbors. Why are these results seemingly discordant? One possibility is that rabies tracing has methodological biases that may not reveal the true synaptic connectivity differences between cell types.^66^ Alternatively, rabies tracing may give a distorted view of overall synaptic input, since it quantifies the number of presynaptic neurons rather than the overall number of synaptic connections (though these might be expected to relate to one another). Using rabies tracing, we quantified the number of presynaptic neurons projecting to a cell type of interest (dMSNs, iMSNs, or TRAPed neurons). However, a single presynaptic neuron may make profuse contacts onto a single or multiple postsynaptic targets,^67^ explaining the functional markers of high synaptic connectivity (reduced PPR and high mEPSC frequency) we observed in TRAPed neurons, as well as the increased amplitude of optically evoked responses onto TRAPed neurons. This pattern of connectivity could drive selective but exceptionally high firing during dyskinesia we observed in TRAPed dMSNs *in vivo*. Using similar methods, another group has identified increased corticostriatal connectivity in the context of cocaine sensitization.^68^

### Limitations of the study

The design of our current study, however, has several limitations. We used a genetic approach (FosTRAP^CreER^) to capture neurons active during a particular time period. This method has several caveats, but most significantly, the time period of capture is estimated to be 8–12 h, which is much longer than the major behavioral effects (approximately 1–2 h) that we focused on here. This long period of capture means that some TRAPed neurons are likely activated by stimuli or behaviors other than dyskinesia. As FosTRAP^CreER^ relies on cFos activation, which is minimal in the healthy and parkinsonian striatum, we were not able to compare the same subpopulation of neurons across healthy, parkinsonian but levodopa-naive, and chronically levodopa-treated states. Instead, we compared the properties of TRAPed neurons before and after acute dopaminergic signaling and to neighboring unTRAPed neurons in the same state. TRAPed MSNs are predominantly dMSNs, but iMSNs contribute to dyskinesia, as well.^69^ Another significant limitation of our study was the small sample of optically identified TRAPed single-units (*n* = 12), which resulted from a combination of their low abundance, a high bar for inclusion of optically labeled units, and the relatively low-yield fixed optrode arrays that we used in this study. A larger dataset would be needed to better assess distinct patterns of activity across this population.

We also focused largely on excitatory input, as this is a key driver of MSN firing. However, motivated by our rabies tracing experiments showing increased proportion of inhibitory GPeMSN inputs in parkinsonian/levodopa-treated mice, which were even more pronounced in TRAPed neurons, we also measured the strength of inhibitory GPe inputs onto dMSNs. While these recordings showed no significant difference between TRAPed and unTRAPed dMSNs, other alterations in the strength or sources of inhibition,^33,70,71^ as well as cholinergic signaling,^72–74^ might also potently shape MSN firing during LID. Future work will need to address the role of these other inputs in shaping striatal activity during LID. Additionally, while we saw a modest increase in the excitability of TRAPed dMSNs following D1R activation, we saw no significant change in unTRAPed dMSN excitability, compared to previous reports of increased excitability following D1R activation in healthy mice.^37–39,75^ This may be driven by two key differences. First, our experiments were performed in the dopamine-depleted, levodopa-treated state, which is known to increase the baseline excitability of dMSNs.^53,76^ This increase in baseline excitability may reduce the effect of dopamine receptor activation seen previously in healthy animals. Second, we used whole-cell, current-clamp recordings to assess excitability. Given that the effect of dopamine receptor activation relies on several downstream signaling cascades, recordings in the whole-cell configuration may dialyze key molecules and blunt the effect of activating dopamine receptors, as has been previously shown in whole-cell compared to perforated-patch recording configurations in healthy mice.^38^

Finally, the differences we observed between TRAPed dMSNs and their neighbors may be related to heterogeneity present in the healthy striatum (fixed factors) or may be related to dopamine-dependent changes that are unevenly distributed across the striatum (plasticity). In fact, healthy striatal subpopulations, defined by receptor expression, specific cortical inputs, or local connectivity, may be differentially vulnerable to the forms of plasticity known to occur in response to chronic dopamine depletion and dopamine replacement. Together, these alterations in excitability or the strength and pattern of inputs may subserve homeostasis in the healthy brain or aid compensation in disease states, but when pushed to their limits, they drive aberrant circuit function and behavior as are seen in LID.

## STAR★METHODS

### RESOURCE AVAILABILITY

#### Lead contact

Further information and requests for resources and reagents should be directed to and will be fulfilled by the lead contact, Alexandra Nelson (Alexandra.nelson@ucsf.edu).

#### Materials availability

This study did not generate any new unique materials.

#### Data and code availability

All data and code generated from this publication are available on Zenodo, BioStudies, and Github (username: UCSF-Nelson-Lab). Any additional information required to reanalyze the data reported in this paper is available from the lead contact upon request.

##### Data

- Zenodo:
  - Data provided for Figures 1, S1, 3, S3, 4, S4, S5, and S6
  - https://doi.org/10.5281/zenodo.10681506
  - Link to Zenodo: https://zenodo.org/records/10681506
- BioStudies
  - Data provided for Figures 2, and S3
  - Link to BioStudies: https://www.ebi.ac.uk/biostudies/studies/S-BSST1374?key=9a1b2103-25a6-49ed-b257-0269d3ea19a6

##### Code

- https://github.com/UCSF-Nelson-Lab/TRAP-Rabies-Analysis.git
- https://github.com/UCSF-Nelson-Lab/TRAP-In-Vivo-Analysis.git
- https://github.com/UCSF-Nelson-Lab/TRAP-RNAscope-Analysis.git
- https://github.com/UCSF-Nelson-Lab/open-field-analysis.git

### EXPERIMENTAL MODEL AND STUDY PARTICIPANT DETAILS

#### Animals

We used 3–9 month old C57Bl/6 mice of either sex. Hemizygous FosTRAP-CreER mice (Liqun Luo, Stanford) were bred to either wild-type C57Bl/6 mice (WT, Jackson Labs) or homozygous Ai14 mice (Jackson Labs) to yield FosTRAP or FosTRAP; Ai14 mice. Hemizygous D2-GFP mice^77^ were bred against WT mice to produce D2-GFP animals. For slice electrophysiology experiments, hemizygous FosTRAP; Ai14 mice were bred to hemizygous D2-GFP mice to yield FosTRAP; Ai14; D2-GFP (FAD) mice. For rabies tracing experiments, Drd1a (line EY217) and Adora2a (line KG139) BAC-Cre mice from the GENSAT Project were used.^78^ Animals were housed 1–5 per cage on a 12-h light/dark cycle with *ad libitum* access to rodent chow and water. All behavioral manipulations were performed during the light phase. We complied with local and national ethical and legal regulations regarding the use of mice in research. All experimental protocols were approved by the UC San Francisco Institutional Animal Care and Use Committee.

### METHOD DETAILS

#### Surgical procedures

A detailed protocol for stereotaxic surgery can be found at dx.doi.org/10.17504/protocols.io.n2bvj6qynlk5/v1. Briefly, all surgical procedures were performed at 3–6 months of age. Anesthesia was induced with intraperitoneal (IP) injection ketamine/xylazine and maintained with 0.5%–1.0% inhaled isoflurane. Mice were placed in a stereotaxic frame and a mounted drill was used to create holes over the left medial forebrain bundle (MFB), the left dorsolateral striatum (DLS), primary motor cortex (M1), primary somatosensory cortex (S1), thalamus, or external globus pallidus (GPe). To render mice parkinsonian, the left MFB (−1.0 AP, +1.0 ML, −4.9 mm DV) was injected using a 33-gauge needle with 1–1.5 μL per site of 6-Hydroxydopamine (6-OHDA)-bromide. In some experiments, AAV5-DIO-ChR2-eYFP (UPenn Vector Core, 1–1.5 μL, diluted 1:3 in NS) was injected in the left DLS (+0.8 AP, +2.3 ML, −2.5 mm DV). For Cre-dependent rabies tracing experiments, 300 nL helper virus, rAAV1/synp-DIO-sTbEpB-GFP (UNC Vector Core, lot AV6118CD) was injected into two left DLS sites (−0.8 AP, −2.4 ML, −2.5 DV). Two weeks after helper virus injection, 300 nL modified EnvA G-deleted Rabies-mcherry (Salk Viral Vector Core) virus was also injected into the same DLS site. For input-specific circuit mapping onto TRAPed cells, 250–300 nL of AAV5-hSyn-hChR2(H134R)-eYFP (UNC Vector Core) was injected into M1 (+1.2 AP, −1.6 ML, −0.7 mm DV), S1 (+0.95 AP, −2.9 ML, −0.75 mm DV), thalamus (−2.3 AP, −0.6 ML, +4.0 mm DV) or GPe (−0.3 AP, −2.2 ML, −4.0 mm DV) in FosTRAP; WT mice. 6-OHDA and virus were injected at a rate of 0.10 μL/min, after which the injection cannula was left in place for 10–15 min prior to being withdrawn and the scalp being sutured.

In preparation for *in vivo* single-unit recordings, FosTRAP; Ai14 mice were injected with 6-OHDA and DIO-ChR2, as described above, and optrode arrays were implanted in a second surgical procedure. After the scalp was reopened, a large craniotomy (1.5 × 1 mm) was created over the left DLS, and two small holes were drilled in the right frontal and right posterior parietal areas for placement of a skull screw (Fine Scientific Tools, FST) and ground wire, respectively. A fixed multichannel electrode array (32 Tungsten microwires, Innovative Neurophysiology) coupled to a 200 μm optical fiber (Thorlabs) was slowly lowered through the craniotomy into the DLS. The final location of the electrode tips was targeted 100–200 μm above the previous DIO-ChR2 injection (−2.3–2.4 mm DV). The array was covered and secured into place with dental cement (Metabond) and acrylic (Ortho-Jet).

All animals were given buprenorphine (IP, 0.05 mg/kg) and ketoprofen (subcutaneous injection, 5 mg/kg) for postoperative analgesia. Parkinsonian animals were monitored closely for 1 week following surgery: mouse cages were kept on a heating pad, and animals received daily saline injections and were fed nutritional supplements (Diet-Gel Recovery Packs and forage/trail mix).

#### Behavior

Postoperatively, parkinsonian mice were monitored in the open field 1–2 times per week for 10 min per session. A detailed protocol can be found at dx.doi.org/10.17504/protocols.io.b9ksr4we. Briefly, all mice were habituated to the open field (clear acrylic cylinders, 25 cm diameter) for 30 min 1–2 days prior to behavioral sessions. Mice were monitored via two cameras, one directly above (to capture overall movement) and one in front of the chamber (to capture fine motor behaviors). Video-tracking software (Noldus Ethovision) was used to quantify locomotor activity, including rotations (90° contralateral or ipsilateral turns), distance traveled, and velocity (see UCSF-Nelson-Lab Github, https://github.com/UCSF-Nelson-Lab/open-field-analysis.git). After a three-week baseline period, mice were injected with levodopa daily for the remainder of the experiment. Levodopa-induced dyskinesia (LID) was scored during weekly sessions in which mice were injected, then placed in a clean, clear cage for visualization. For regular weekly dyskinesia scoring, 1–2 blinded experimenters rated AIMs (for details see Statistical Procedures below). For *in vivo* electrophysiology experiments, rotations and AIMs were quantified in 1-min bins, with dyskinesia being scored every other minute.

#### Pharmacology

6-OHDA (Sigma Aldrich) for MFB dopamine depletions was prepared at 5 μg/μL in normal saline solution. Levodopa (Sigma Aldrich) was administered with benserazide (Sigma Aldrich) and prepared in normal saline solution. Levodopa (5–10 mg/kg) was given via IP injection 5–7 days per week over the course of the experiment. Initially, on the 7^th^ day of levodopa treatment for FosTRAP; WT, FosTRAP; Ai14, and FosTRAP; Ai14; D2-GFP mice were given 4-hydroxytamoxifen (4-OHT, 50 mg/kg in Chen oil, IP) exactly 1 h post-levodopa injection, to capture dyskinesia-associated neurons (Figure 1A). 4-OHT was prepared as previously described.^19,20^ Briefly, to prepare a 20 mg/mL stock in ethanol of 4-OHT, 4-OHT was added to 200 proof ethanol, vortexed, and placed on a horizontal shaker at 37°C for 30 min or until the 4-OHT dissolved. The stock solution was kept covered in foil to minimize light exposure. Next, to prepare a 10 mg/mL working solution in oil, the 4-OHT/ethanol mixture was combined with Chen Oil (a mixture of 4 parts sunflower seed oil and 1 part castor oil) and placed into 1.5 mL Eppendorf tubes. The tubes were vigorously mixed, wrapped in foil, and left on a nutator for 45 min at room temperature, vortexed and shaken periodically. The tubes were then placed in a speed-vac for 2–3 h to evaporate the ethanol. If necessary, the final volume was adjusted with Chen Oil to 1 mL to reach a final concentration of 10 mg/mL. Both levodopa and 4-OHT were injected in a quiet, familiar environment, and animals were returned to their home cages, to minimize additional stimuli. Daily levodopa injections continued for 2–6 weeks to allow expression of Cre-dependent constructs. For *ex vivo* experiments, picrotoxin (Sigma) was dissolved in warm water to prepare a 5 mM stock solution, which was subsequently diluted in ACSF for a final concentration of 50 μM. Tetrodotoxin (TTX, Abcam) was dissolved in water at a stock concentration of 1 mM and added to ACSF for a final concentration of 1 μM. SKF 81298 (Tocris) was dissolved in water at a concentration of 1mM and added to ACSF for a final concentration of 5 μM. For all *ex vivo* experiments, biocytin (1–2.5 mg/mL) was included in the internal solution for post-hoc confirmation of the presence or absence of Ai14 and D2-GFP.

#### *In vivo* electrophysiology

A detailed protocol for *in vivo* electrophysiology, including optrode array fabrication and data acquisition, can be found at dx.doi.org/10.17504/protocols.io.5jyl89w69v2w/v1. Two weeks after optrode array implantation, mice were habituated to tethering and the recording chamber for 1–2 days. After habituation, experimental sessions occurred 3–5 times per week for 2–6 weeks. During each session, electrical signals (single-unit and LFP data from each of 32 channels) were collected using a multiplexed 32 channel headstage (Triangle Biosystems), an electrical commutator equipped with a fluid bore (Dragonfly), filtered, amplified, and recorded on a MAP system, using RASPUTIN 2.4 HLK3 acquisition software (Plexon). Spike waveforms were filtered at 154–8800 Hz and digitized at 40 kHz. The experimenter manually set a gain and threshold for storage of electrical events.

During recording sessions, after a baseline period of 30 min in the parkinsonian state, levodopa (5–10 mg/kg) was injected IP. After a period of 2–3 h of recording spontaneous activity in the open field, an optogenetic cell identification protocol was applied^20^ consisting of 100 msec blue light pulses, given at 1 Hz. At each of 4 light powers (0.5, 1, 2, and 4 mW), 1000 light pulses were delivered via a lightweight patch cable (Doric Lenses) connected to a blue laser (Shanghai Laser and Optics Century), via an optical commutator (Doric Lenses), and controlled by TTL pulses from a behavioral monitoring system (Noldus Ethovision).

Single-units were identified offline by manual sorting using Offline Sorter 3.3.5 (Plexon) and principal components analysis (PCA). Clusters were considered to represent a single unit if (1) the unit’s waveforms were statistically different from multiunit activity and any other single-units on the same wire, in 3D PCA space, (2) no interspike interval <1 msec was observed. Single-units were then classified as putative medium spiny neurons (MSNs) or interneurons (INs) as previously described^25,26,79^ using features of the spike waveform (peak to valley and peak width), as well as inter-spike interval distribution.

After single-units had been selected for further study, their firing activity was analyzed using NeuroExplorer 4.133 (Nex Technologies). To determine if a unit was optogenetically identified, a peristimulus time histogram was constructed around the onset of laser pulses. To be considered optogenetically identified, a unit had to fulfill 3 criteria: (1) the unit had to increase firing rate above the 99% confidence interval of the baseline within 15 msec of laser onset; (2) the unit’s firing was above this threshold for at least 15 msec; (3) the unit’s laser-activated waveforms were not statistically distinguishable from spontaneous waveforms.

#### *Ex vivo* electrophysiology

A detailed protocol for slice electrophysiology can be found at dx.doi.org/10.17504/protocols.io.6qpvr67rpvmk/v1. Prior to terminal anesthesia and preparation of brain slices, animals (3–9 months) were co-injected with levodopa and benserazide (5–10 mg/kg and 2.5–5 mg/kg, respectively) to induce LID. After 30–45 min in the dyskinetic state, mice were deeply anesthetized with an IP ketamine-xylazine injection, transcardially perfused with ice-cold glycerol-based slicing solution, decapitated, and the brain was removed. The glycerol-based slicing solution contained (in mM): 250 glycerol, 2.5 KCl, 1.2 NaH_2_PO_4_, 10 HEPES, 21 NaHCO_3_, 5 glucose, 2 MgCl_2_,2 CaCl_2_. The brain was mounted on a submerged chuck, and sequential 275 μm coronal slices were cut on a vibrating microtome (Leica), transferred to a chamber of warm (34°C) carbogenated ACSF containing (in mM) 125 NaCl, 26 NaHCO_3_, 2.5 KCl, 1 MgCl_2_, 2 CaCl_2_, 1.25 NaH_2_PO_4_, 12.5 glucose for 30–60 min, then stored in carbogenated ACSF at room temperature. Each slice was then submerged in a chamber superfused with carbogenated ACSF at 31°C–33°C for recordings.

Striatal medium spiny neurons were targeted for recordings using differential interference contrast (DIC) optics in FosTRAP; Ai14; D2GFP mice on a Olympus BX 51 WIF microscope. In FosTRAP; Ai14; D2GFP mice, TRAPed neurons were identified by their tdTomato-positive somata and D2-positive neurons were identified by GFP fluorescence. Fluorescence-negative neurons with GABAergic interneuron physiological properties (membrane tau decay <0.8 ms for both fast-spiking and persistent low-threshold spiking subtypes; input resistance >500 MΩ in persistent low-threshold spiking subtype) were excluded from the analysis.

Neurons were patched in whole-cell voltage-clamp configurations using borosilicate glass electrodes (3–5 MΩ) filled with cesium-based (voltage-clamp) or potassium methanesulfonate-based (current-clamp) internal solution. A cesium-based solution was used to measure inward excitatory postsynaptic currents (EPSCs) at a holding potential of −70 mV. It contained (in mM): 120 CsMeSO_3_, 15 CsCl, 8 NaCl, 0.5 EGTA, 10 HEPES, 2 MgATP, 0.3 NaGTP, 5 QX-314, pH 7.3. A cesium-based solution with higher chloride was used to measure inward inhibitory postsynaptic currents (IPSCs) at a holding potential of −70 mV. It contained (in mM): 15 CsMeSO_3_, 120 CsCl, 8 NaCl, 0.5 EGTA, 10 HEPES, 2 MgATP, 0.3 NaGTP, 5 QX-314, pH 7.3. A potassium-based solution was used to measure intrinsic excitability. It contained (in mM): 130 KMeSO_3_, 10 NaCl, 2 MgCl_2_, 0.16 CaCl_2_, 0.5 EGTA, 10 HEPES, 2 MgATP, 0.3 NaGTP, pH 7.3. For recordings of intrinsic excitability and/or EPSCs, picrotoxin (50 μM) was added to the external solution to block synaptic currents mediated by GABA_A_ receptors. Drugs were prepared as stock solutions and added to the ACSF to yield the final concentration.

Whole-cell recordings were made using a MultiClamp 700B amplifier (Molecular Devices) and ITC-18 A/D board (HEKA). Data was acquired using Igor Pro 6.0 software (Wavemetrics) and custom acquisition routines (mafPC, courtesy of M. A. Xu-Friedman). Both voltage clamp and current-clamp recordings were filtered at 2 kHz and digitized at 10 kHz. All recorded neurons exhibited electrophysiological characteristics of medium spiny neurons. All synaptic currents were recorded with a cesium-based internal and monitored at a holding potential of −70 mV. Series resistance and leak currents were monitored continuously. Miniature EPSCs were recorded at −70 mV in 1 μM TTX and 50 μM picrotoxin. Evoked EPSCs onto medium spiny neurons were elicited in the presence of picrotoxin (50 μM) with a stimulus isolator (IsoFlex, AMPI) and a glass electrode placed dorsolateral to the recorded neuron, typically 100–200 μm away. Stimulus intensity was adjusted to yield EPSC amplitudes of approximately 400 pA with a stimulus duration of 300 μs. For evaluation of the paired pulse ratio, two stimuli were given at variable interstimulus intervals (ISIs; 25, 50, 100, 200, 500 ms) with a 20 s intertrial interval. Paired-pulse ratio is defined as EPSC_2_/EPSC_1_. Five to eight repetitions at each ISI were averaged to yield the PPR for that ISI. For monitoring of EPSC amplitude over time, two pulses delivered with 50 ms interstimulus interval were given every 20 s. For AMPA/NMDA ratio experiments, one stimulus at −70 mV or +40 mV was given every 20 s, at 15–20 repetitions per holding potential. AMPA/NMDA ratios were calculated as the ratio of the magnitude of the EPSC at +40 mV at 50 ms following stimulation (NMDA) to the peak of the EPSC at −70 mV (AMPA). ChR2-mediated synaptic currents from M1, S1, thalamus, or GPe were optically evoked using 2 ms pulses of 473 nm light at light powers of 0.5, 1, 2, and 4 mW and delivered by a TTL-controlled LED (Olympus) passed through a GFP filter (Chroma). To isolate excitatory responses, M1, S1, and thalamic stimulation was performed in the presence of picrotoxin (picrotoxin was omitted for GPe stimulation).

Current-clamp recordings were made to measure the intrinsic properties of striatal neurons. The resting membrane potential (V_m_) was measured as the average V_m_ 5–10 min after break-in. A series of small negative current steps were delivered from rest to calculate the input resistance of each cell. Rheobase and other input-output properties were obtained by giving a series of square-wave current steps, ranging from 100 pA to 600 pA, in 100 pA increments, with a 10 s interstimulus interval. Drugs, such as SKF-81297, were applied after achieving a stable baseline 5–10 min after break-in. Changes in intrinsic properties due to SKF were assessed 10–15 min after drug wash-in.

#### Monosynaptic rabies tracing

A detailed protocol for monosynaptic rabies tracing can be found at dx.doi.org/10.17504/protocols.io.3byl4qp9jvo5/v1. All D1-Cre and A2a-Cre mice were used to perform monosynaptic retrograde tracing onto direct and indirect pathway neurons, respectively. Groups of healthy (non-depleted), parkinsonian, and parkinsonian/levodopa-treated mice were used within each genotype. Mice were rendered parkinsonian as described above. Four weeks after dopamine depletion, animals received daily injections of levodopa. Parkinsonian mice (one week into daily levodopa injections) or untreated healthy mice, were then anesthetized and a Cre-dependent helper virus (AAV1-DIO-sTpEpB-GFP) was stereotaxically injected into the left DLS (ipsilateral to the depletion in parkinsonian mice). The helper virus (AAV1-DIO-sTpEpB-GFP) expresses the EnvA receptor (TVA) and rabies glycoprotein necessary for rabies infection and replication in a cell-type specific manner, termed “starter cells,” which are labeled with the green fluorophore GFP. After animals recovered for two weeks, they were anesthetized, and a replication-incompetent form of the rabies virus (EnvA-G-deleted-rabies-mCherry) was stereotaxically injected into the DLS using the same coordinates. The rabies virus will then infect a subset of starter cells (co-infected) and travel retrogradely one synapse, expressing the red fluorophore mCherry in infected cells. Once the rabies virus infects a presynaptic neuron, uninfected with the helper virus, it will no longer be capable of replication and/or retrograde synaptic infection. Rabies injections were performed in an approved Biosafety Level 2 (BSL-2) surgical suite. After animals recovered for ten days, they were terminally anesthetized with ketamine/xylazine (200/40 mg/kg I.P.), transcardially perfused with 4% paraformaldyde (PFA), and the brain dissected from the skull. Brains were post-fixed overnight in 4% PFA and then placed in 30% sucrose at 4°C.

Parkinsonian, levodopa-treated FosTRAP; WT mice were prepared in a similar fashion as D1-Cre and A2a-Cre mice, with some alterations made to the experimental timeline to accommodate helper virus expression using the conditional Cre (CreER) in the FosTRAP line. In FosTRAP mice, helper virus was injected in the left DLS at the same time as the initial dopamine depletion. Three weeks after dopamine depletion, FosTRAP mice began daily levodopa injections. After one week of daily levodopa injections, as above, FosTRAP mice were injected with levodopa followed by an injection of 4-OHT, allowing for recombination and expression of the helper virus. Two weeks later, FosTRAP mice were anesthetized and the modified rabies virus was injected using the same procedures described above. The remainder of the experimental timeline was similar to that for D1-Cre and A2a-Cre mice as described above.

Fixed brains, stored in sucrose, were then sent to Dr. Charles Gerfen at the National Institutes of Mental Health (NIMH) for sectioning, mounting, imaging, and analysis using published methods.^29^ Briefly, brains were sectioned coronally at 50 μm using a freezing microtome. Sections were processed for fluorescent immunohistochemical localization of GFP labeling of rabies starter cells, RFP labeling of transsynaptically transported rabies, and tyrosine hydroxylase to label the nigrostriatal dopamine system. Slices were then imaged using a Zeiss microscope equipped with a z axis drive, imaging each fluorophore. The imaged coronal sections were reconstructed into a whole brain volume, labeled cells detected using a modified Laplacian of Gaussian algorithm and then registered to the Allen Common Coordinate mouse atlas framework using NeuroInfo software (MBF Biosciences, Williston, VT). In a subset of FosTRAP brains, slices were additionally stained for Npas1 (primary antibody: provided courtesy of the laboratory of Savio Chan at Northwestern^34^; secondary antibody: Goat anti-Guinea Pig IgG, Alexa Fluor 647, Life Technology: A21450) and PV (primary antibody: mouse anti-PV, Millipore MAB1572; secondary antibody: Goat anti-Mouse IgG, DyLight 755, Life Technology: SA5–10175) to assess the molecular identify of rabies-labeled presynaptic GPe neurons (Figures S5A–S5D).

#### Histology & Microscopy

A detailed protocol for histological processing can be found here: https://www.protocols.io/view/immunohistochemistry-14egn7nezv5d/v1. After rabies tracing or behavioral experiments, mice were deeply anesthetized with IP ketamine-xylazine and transcardially perfused with 4% paraformaldehyde in PBS. Following *in vivo* electrophysiology experiments, prior to perfusion, electrode array location was marked by electrolytic lesioning. After deep anesthesia, the implant was connected to a solid state, direct current (DC) Lesion Maker (Ugo Basile). A current of 100 μA was passed through each microwire for 5 s. After perfusion, the brain was dissected from the skull and post-fixed overnight in 4% paraformaldehyde, then placed in 30% sucrose at 4°C for cryoprotection. The brain was then cut into 35 μm coronal or sagittal sections on a freezing microtome (Leica) and then mounted in Vectashield Mounting Medium onto glass slides for imaging. For immunohistochemistry, the tissue was blocked with 3% normal donkey serum (NDS) and permeabilized with 0.1% Triton X-100 for 2 h at room temperature on a shaker. Primary antibodies were added to 3% NDS and incubated overnight at 4°C on a shaker. Primary antibodies used: Rabbit anti-TH (Pel-Freez, 1:1000), Chicken anti-TH (Sigma, 1:1000), and Chicken anti-GFP (1:500). Slices were then incubated in secondary antibodies (donkey anti-rabbit or chicken Alexa Fluor 488, 593, or 647, 1:500, JacksonImmuno Research) for 2–4 h at 4°C on a shaker, washed, and mounted onto slides for imaging. 4 or 10x images were acquired on a Nikon 6D conventional widefield microscope.

For slice electrophysiology experiments in which the internal solution contained biocytin, slices were subsectioned at 50 μm and washed in PBS. Slices were blocked for 2 h at room temperature on a shaker in a 5% NDS and 0.3% Tween 20 PBS-based solution. Primary antibodies were the same as described above. Slices were then incubated in secondary antibodies (donkey anti-rabbit or chicken Alexa Fluor 488, 593, or 647, 1:500, JacksonImmuno Research and Streptavidin Alexa 350, 3:500, Sigma) for 6–12 h at 4°C on a shaker, washed, and mounted onto slides for imaging. Images were acquired on a Nikon 6D conventional widefield or Nikon Spinning Disk confocal microscope with a 40x objective microscope. Exposure times were matched between images of the same type. Post-hoc confirmation of cellular identify of a subset of recovered biocytin-filled, recorded cells revealed that online identification by experimenter using fluorescence intensity was >90% in FosTRAP; Ai14; D2-GFP mice for TRAPed dMSNs and unTRAPed dMSNs and iMSNs. The rate of positive identification of TRAPed and unTRAPed dMSNs in FosTRAP:Ai14; D2-GFP mice injected with ChR2-eYFP in M1, S1, thalamus, and GPe was also >90%, however, due to the overlap of YFP and GFP emission spectra, our positive rates of identification of unTRAPed iMSNs was reduced to ~60%, leading to the exclusion of unTRAPed iMSNs from these experiments (Figures 3E–3P, and S5E–S5H).

#### Fluorescent *In situ* hybridization

A detailed protocol for RNAscope methodology can be found at https://dx.doi.org/10.17504/protocols.io.14egn3odml5d/v1. To compare the expression of several key mRNA transcripts in cell types of the DLS, we treated FosTRAP; Ai14 mice with unilateral 6-OHDA and chronic IP levodopa (5 mg/kg), as described above. 4-OHT was administered on day 8 of levodopa. After 2 additional weeks of daily levodopa treatment, animals were sacrificed (2 h after the last levodopa injection) and transcardially perfused, as described above in Histology and Microscopy. Dissection instruments were sprayed before and between animals with RNaseZap, and solutions were prepared with RNase-free distilled water. After 24 h in 4% paraformaldehyde, brains were transferred to 30% sucrose, and subsequently prepared for RNAscope. Brains were then cryosectioned at 20μm from AP +1.0 to 0.0 in RNase-free PBS and mounted onto slides. RNAscope Multiplex fluorescent *in situ* hybridization was then performed to stain for D1 dopamine receptor (RNAscope Probe Mm-Drd1-C2, ref. 461901-C2), D2 dopamine receptor (RNAscope Probe Mm-Drd2-C3, ref. 406501-C3), prodynorphin (RNAscope Probe Mm-Pdyn-C3, ref. 318771-C3), and TRAP-tdTomato mRNA (RNAscope Probe tdTomato, ref. 317041). Fluorescent dyes were then used to visualize separate probe channels. In every section, D1 dopamine receptors and TRAP-tdTomato were visualized using TSA Vivid Fluorophore 520 (ref. 323271) and TSA Vivid Fluorophore 570 (ref. 323272), respectively. Each section was also stained for either D2 dopamine receptors or pDyn, which was visualized using TSA Vivid Fluorophore 650 (ref. 323273). Slides were then imaged with a Zeiss Axioscan 7 slide scanner at 20x magnification (Plan-Apochromat 20x/0.8 M27 Objective) using a z stack and maximum intensity projection.

### QUANTIFICATION AND STATISTICAL ANALYSIS

#### Statistics

All details can be found in the Table S1. All data are presented as the mean ± SEM, with “N” referring to the number of animals and “n” to the number of cells or slices. Goal sample sizes for physiological studies were chosen using a power calculation, with a two-sided alpha of 0.05, power of 0.9, and the statistical tests listed below under each section. For behavioral and *in vivo* physiology assays, goal sample size was driven by the lowest-yield component of the experiments (optogenetically-labeled single-unit recordings of TRAPed neurons, goal *n* = 10). The power calculation relied on estimates of effect size from DYSK vs. ON dMSN subtypes.^15^ For *ex vivo* physiology assays, the power calculation relied on previously acquired data in the lab, in another mouse model of dyskinesia,^80^ to estimate average, standard deviation, and effect size. Power calculations yielded *n* = 15 cells/cell type per group for mEPSCs, and *n* = 10 cells/group for evoked EPSC analyses. For *ex vivo* pharmacology, we again used previously acquired data in the lab,^15,20,80^ as well as published data on the effects of D1 agonists^39^ to calculate the sample size, which yielded *n* = 7 cells per group. For all *ex vivo* electrophysiology, regardless of the minimum n (cells), we had a goal of *N* > 4 (mice). For modified rabies tracing, there were few precedents in the literature to inform a power calculation, so we chose a goal sample size of *N* = 5 mice per group, per cell type. For fluorescent *in situ* hybridization experiments, based on precedent from the literature, we chose a goal sample size of *N* = 5 mice per group.

#### Behavior

Dyskinesia was quantified using a standard scoring method,^21^ which takes into account abnormal involuntary movements (AIMs) in axial, limb, and orofacial (ALO) body segments. Briefly, dyskinesia was quantified every 20 min, over a 2-h period, using a scale of 0–4. A score of 0 indicates no abnormal movement, and a score of 4 describes continuous and uninterruptible dyskinetic movements; 12 (4 × 3 body segments) is the maximum score possible for a given time point. Dyskinesia was quantified every other minute during *in vivo* electrophysiology experiments.

#### *Ex vivo* electrophysiology

For excitability, current-response curves (Figures 4A–4H) were compared using a one-way repeated measures ANOVA, either across cell-types (Figure 4E) or within a cell-type before and after application of SKF-81297 (Figures 4F–4H), with a post-hoc Tukey test. Passive and active properties across the three cell-types were compared using a nonparametric Kruskal-Wallis (KW) test, with a posthoc Tukey test (Table S2) or within cell-types before and after application of SKF-81297 using a paired, nonparametric Wilcoxon signed-rank test (Table S3). Significant *p*-values were determined following Bonferroni correction for multiple comparisons. Frequency and amplitude of mESPCS (Figures 3A, 3B, S4A, and S4B), as well as AMPA/NMDA ratio (Figures S4C and S4D) were compared between the three cell-types using a KW test. Paired-pulse ratio curves (Figures 3C and 3D) were compared between the three cell-types using a one-way repeated measures ANOVA, with a posthoc Tukey test. For mEPSC frequency and amplitude measurements, only cells with at least 500 detected events were included in subsequent analysis. Cumulative probability plots were generated from 500 randomly selected mEPSC events per cell. Changes in excitability in response to acute SKF application were analyzed by comparing a 10-min baseline period with the value 10–15 min after drug application. Average amplitudes of oEPSCs were quantified manually in Igor. A nonparametric Wilcoxon sign-rank (SR) test was used to compare oEPSC amplitudes from M1, S1, or thalamus onto TRAP vs. unTRAP dMSNs, and to compare oIPSC amplitudes from GPe onto TRAP vs. unTRAP dMSNs. In all experiments involving optical stimulation, data was drawn from stimulations at 0.5, 1, 2,4 mW, with subsequent statistical comparisons being made at 4 mW (Figures 3J, 3M, 3P, and S5G).

#### *In vivo* electrophysiology

For most analyses of single-unit firing rate and behavior, firing rate was averaged in 1-min bins. Modulation of firing rate by levodopa was determined by comparing single-unit firing rates before and after drug administration, during the peak behavioral effects. The 30-min baseline period was compared to a 30-min period following drug injection (10–40 min post-injection). Following levodopa administration, unlabeled single-units were categorized into three broad groups as follows, based on significant changes in firing rate (*p* < 0.01, Wilcoxon rank-sum test (RS)) following levodopa treatment: putative dMSNs (On MSNs, increase in firing rate), putative iMSNs (Off MSNs, decrease in firing rate), or no change units (NC, nonsignificant change in firing rate) (Figure 1G). For levodopa sessions, putative dMSNs were further divided using behavior-based methods, as described previously.^15^ For the behavior-based method, AIM scores were also averaged in 1-min bins and correlated with firing rate using linear regression. Labeled TRAPed neurons or putative dMSNs with a significant correlation (R^2^ > 0.30) to AIM score were labeled dyskinesia (DYSK) units and those with no significant correlation (R^2^ < 0.30) to AIMs were classified as on-unclassified (ON) units (Figures 1L–1O, and S1P).

Firing rates of parkinsonian mice before (Park) and after drug administration (levodopa (LID), Figure 1K) were compared between optogenetically labeled TRAPed putative dMSNs and all putative dMSNs using Wilcoxon rank-sum test (RS). Comparisons between the average dyskinesia correlation of optogenetically labeled TRAPed and all putative dMSNs were made using Wilcoxon rank-sum test (RS).

#### Monosynaptic rabies tracing

Custom analyses were written in MATLAB for quantification of rabies labeled cells (see UCSF-Nelson-Lab Github for code, https://github.com/UCSF-Nelson-Lab/TRAP-Rabies-Analysis.git). To ensure consistent striatal labeling across mice, an inclusion criterion of <15% spread of “starter” cells (sTpEpB, green) outside of the striatum was applied to all brains before proceeding to subsequent quantification (Figure S3B). The relative number of presynaptic neurons was quantified by dividing the total number of presynaptic neurons in the specified brain region by the total number of co-infected (sTpEpB, green and rabies, red) striatal neurons (Figure S3D). The relative proportion of presynaptic neurons was then quantified by dividing the total number of presynaptic neurons in the specified brain region by the total number of presynaptic (rabies-labeled, red) extra-striatal neurons detected in the whole brain. Results were when pooled across mice of the same genotype (D1-Cre, A2a-Cre, or TRAP-CreER) and treatment condition (control, levodopa-naïve parkinsonian, or levodopa-treated parkinsonian). Comparisons between treatments conditions for iMSNs and dMSNs were compared using a nonparametric Kruskal-Wallis test, with posthoc Tukey test (Figures 2 and S3). Comparisons between TRAP-CreER and D1-Cre levodopa-treated, parkinsonian mice were made using a Wilcoxon rank-sum test (RS).

#### Fluorescent In situ hybridization

RNAscope sections were viewed and quantified using QuPath software. Annotations were made in the ispilesional striatum in the DLS, DMS, and VLS (Figure S6C) for subsequent quantification of TRAP-tdTomato, D1R, D2R, and pDyn mRNA expression. First, D1R + cells (putative dMSNs) were identified using the Cell Detection feature in QuPath for the Drd1a probe channel, using the same setup and nucleus parameters across sections, with the intensity threshold for detection adjusted per slice to account for differences in staining intensity across slices. Once D1R + cells were identified, a single measurement classifier was used to identify TRAPed cells using an intensity threshold for the TRAP-tdTomato probe channel. Fluorescence and cell size measurements for all probe channels (D1R, D2R, pDyn) for all D1R + cells were then exported and saved as a csv file.

Custom analyses were written in MATLAB to normalize and quantify fluorescence intensity values for each probe across cells (see UCSF-Nelson-Lab Github for code, https://github.com/UCSF-Nelson-Lab/TRAP-RNAscope-Analysis.git). To compare mRNA expression levels across cells, we first performed a “cell” normalization by dividing the sum of all pixel intensities for each cell by its area to account for differences in cell size/shape. Next, we performed a “slice” normalization by dividing these cell-normalized values by the mean of all cells in the slice, either pooled across the striatum (Figures 4K–4M) or for each subregion (DLS, DMS, or VLS; Figures S6C–S6E). This slice normalization accounted for variation in overall probe intensity across slices and subregions. Comparisons between TRAPed and unTRAPed dMSNs were made for each probe (D1R, D2R, or pDyn) using a Wilcoxon signed-rank (SR) test (Figures 4K–4M). Comparisons between TRAPed and unTRAPed dMSNs in striatal subregions (DLS, DMS, VLS) for D1R and pDyn probes were made using a Wilcoxon signed-rank (SR) test, correcting for multiple comparisons (Figures S6C–S6E).

## Supplementary Material

1

## Figures and Tables

**KEY RESOURCES TABLE:** 

REAGENT or RESOURCE	SOURCE	IDENTIFIER

Antibodies		

Rabbit anti-TH	Pel-Freez	RRID: AB_2617184; Cat#P40101-150
Chicken anti-TH	Millipore	RRID: AB_570923; Cat#AB9702
Chicken anti-GFP	Abcam	RRID: AB_300798; Cat#ab13970
Guinea pig anti-Npas1	Savio Chan	Ref. 34; Avail on Request
Mouse anti-PV	Millipore	RRID: AB_2174013; Cat#MAB1572
Alexa Fluor 488 Donkey Anti-Rabbit IgG	Jackson ImmunoResearch	RRID: AB_2340619; Cat#711-546-152
Alexa Fluor 568 Donkey Anti-Rabbit IgG	Invitrogen	RRID: AB_2534017; Cat#A10042
Alexa Fluor 647 Donkey Anti-Rabbit IgG	Jackson ImmunoResearch	RRID: AB_2340625; Cat #711-606-152
Alexa Fluor 488 Donkey Anti-Chicken IgY (IgG)	Jackson ImmunoResearch	RRID: AB_2340375; Cat #703-545-155
Alexa Fluor 647 Donkey Anti-Chicken IgY (IgG)	Jackson ImmunoResearch	RRID: AB_2340380; Cat#703-606-155
Alexa Fluor 647 Goat Anti-Guinea Pig IgG	Life Technology	RRID: AB_2735091; Cat#A21450
DyLight 755 Goat Anti-Mouse IgG	Life Technology	RRID: AB_2556755; Cat#SA5-10175
RNAscope Probe Mm-Drd1-C2	Acdbio	461901-C2
RNAscope Probe Mm-Drd2-C3	Acdbio	406501-C3
RNAscope Probe Mm-Pdyn-C3	Acdbio	318771-C3
RNAscope Probe tdTomato	Acdbio	317041
TSA Vivid Fluorophore 520	Acdbio	323271
TSA Vivid Fluorophore 570	Acdbio	323272
TSA Vivid Fluorophore 650	Acdbio	323273

Bacterial and virus strains		

AAV5-EF1a-DIO-eYFP	UNC Vector Core	RRID: Addgene_27056; Lot#AV4310g
AAV5-EF1a-DIO-hChR2(H134R)-eYFP-WPRE-hGH	Penn Vector Core	RRID: Addgene_20298; Lot #CS0384
AAV5-hSyn-ChR2(H134R)-eYFP	UNC Vector Core	RRID: Addgene_26973
AAV1-synP-DIO-sTpEpB	UNC Vector Core	RRID: Addgene_52473; Lot#AV6118CD
EnvA-G-deleted-rabies-mCherry	Salk Vector Core	RRID: Addgene_32636

Chemicals, peptides, and recombinant proteins		

Picrotoxin	Sigma-Aldrich	P1675
Lidocaine N-ethyl chloride	Sigma-Aldrich	L1663
6-Hydroxydopamine hydrobromide	Sigma-Aldrich	H116
Potassium Methanesulfonate	Sigma-Aldrich	83000
Guanosine 5’-triphosphate sodium salt	Sigma-Aldrich	G8877
Adenosine 5’-triphosphage magnesium salt	Sigma-Aldrich	A9187
Despiramine hydrochloride	Sigma-Aldrich	D3900
Benserazide hydrochloride	Sigma-Aldrich	B7283
SKF 81297 hydrobromide	Tocris	1447
3,4-Dihydroxy-L-phenylalanine	Sigma-Aldrich	D9628
Cesium methanesulfonate	Sigma-Aldrich	C1426
CNQX	Tocris	1045
D-APV	Tocris	106
Triton X-100	Sigma-Aldrich	T8787
Biocytin	Sigma-Aldrich	B4261
RNase Zap	Sigma-Aldrich	R2020

Critical commercial assays		

VECTASHIELD Antifade Mounting Medium	Vector Laboratories	RRID: AB_2336789; Cat#H-1000

Experimental models: Organisms/strains		

Mouse: Wild-type C57Bl/6J	The Jackson Laboratory	RRID: IMSR_JAX:000664
Mouse: B6.129(Cg)-Fostm1.1(creERT2)Luo/J	The Jackson Laboratory	RRID: IMSR_JAX:021882
Mouse: Stock Tg(Drd2-EGFP)S118Gsat/Mmnc Mus Musculus	MMRRC	RRID: MMRRC_000230-UNC
Mouse: B6.Cg-Gt(ROSA)26Sortm14(CAG-tdTomato)Hze/J	The Jackson Laboratory	RRID: IMSR_JAX:007914
Mouse: B6.FVB(Cg)-Tg(Drd1-Cre)EY217Gsat/Mmucd	MMRRC	RRID: MMRRC_034258-UCD
Mouse: B6.FVB(Cg)-Tg(Adora2a-Cre)KG139Gsat/Mmucd	MMRRC	RRID: MMRRC_036158-UCD

Software and algorithms		

IgorPro	Wavemetrics	RRID: SCR_00325; http://www.wavemetrics.com/products/igorpro/igorpro.htm
mafPC (software package for use with IgorPro)	Xu-Friedman Lab	https://www.xufriedman.org/mafpc
MATLAB R2015a	MathWorks	RRID: SCR_001622; https://www.mathworks.com/products/matlab/
ImageJ	NIH	RRID: SCR_003070; https://imagej.nih.gov/ij/
EthovisionXT	Noldus	RRID: SCR_000441; http://www.noldus.com/animal-behavior-research/products/ethovision-xt
Adobe Illustrator CS5	Adobe	RRID: SCR_014198; http://www.adobe.com/products/illustrator
MAP Software (RASPUTIN 2.4)	Plexon	RRID: SCR_003170; https://plexon.com/wp-content/uploads/2017/06/RASPUTIN-Manual.pdf
Offline Sorter	Plexon	RRID: SCR_000012; http://plexon.com/products/offline-sorter
NeuroExplorer	Plexon	RRID: SCR_001818; http://www.neuroexplorer.com/
Axon MultiClamp Commander	Axon	RRID: SCR_018455; http://mdc.custhelp.com/app/answers/detail/a_id/18877/~/axon%E2%84%A2-multi
NIS-Elements	Nikon	RRID: SCR_014329; https://www.microscope.nealthcare.nikon.com/products/soffware
NeuroInfo	MBF Biosciences	RRID: SCR_017346; https://www.mbfbioscience.com/products/neuroinfo/
QuPath	QuPath	RRID: SCR_018257; https://qupath.github.io/
Other		
32 Channel Fixed Optrode Array	Innovative Neurophysiology	Custom; http://www.inphysiology.com/optogenetic-applications/
200 mm Core TECS-Clad Multimode Optical Fiber, 0.39 NA	Thorlabs	Cat#FT200UMT
1.25 mm Multimode LC/PC Ceramic Ferrule, 230 mm Bore Size	Thorlabs	Cat#CFLC230-10
Ceramic Split Mating Sleeve for 1.25 mm (LC/PC) Ferrules	Thorlabs	Cat#ADAL1
150mW DPSS 473nm Blue Laser	Shanghai Laser and Optics Century	Cat#BL473T8-150 + ADR-800A
200 mm Core, 0.39 NA FC/PC to 01.25 mm Ferrule Patch Cable, 1 m Long	Thorlabs	Cat#M83L01
1x1 Fiber-optic Rotary Joint	Doric Lenses	Ca# FRJ_1x1_FC-FC
1x2 Fiber-optic Rotary Joint	Doric Lenses	Ca# FRJ_1x2i_FC-2FC_0.22
10-Channel Slip Ring Electrical Commutator	Dragonfly	Model SL-10-C; https://campdeninstruments.com/products/10-ch-slip-ring-commuta
Master-8	AMPI	http://www.ampi.co.il/master8cp.html
M-Series (M60) Amplifier System for Multiplexing	Triangle BioSystems	http://www.trianglebiosystems.com/m-series-systems.html
Single Channel Temperature Controller	Warner Instruments	Cat#TC-324C
MINIPULS 3 Peristaltic Pumps	Gilson	Cat#F155008
X-Cite 120LED Boost	Excelitas	Cat#010-00326R
ITC-18 16-bit Multi-Channel Data Acquisition Interface	Heka	RRID: SCR_023164
Multiclamp 700B Microelectrode Amplifier	Molecular Devices	RRID: SCR_018455

## Highlights

- FosTRAP captures striatal neurons activated in levodopa-induced dyskinesia
- Levodopa evokes high firing rates in TRAPed direct pathway striatal neurons (dMSNs)
- TRAPed dMSNs show enhanced dopamine sensitivity and excitatory synaptic input
