# Supplementary material for: Excessive firing of dyskinesia-associated striatal direct pathway neurons is gated by dopamine and excitatory synaptic input
Source: Cell Rep. Author manuscript; Available in PMC 2025 May 15. (PMC12080562; doi:10.1016/j.celrep.2024.114483)
Supplement: 1 [file NIHMS2076378-supplement-1.pdf]

Cell Reports, Volume *43*

Supplemental information

Excessive firing of dyskinesia-associated striatal direct pathway neurons is gated by dopamine and excitatory synaptic input

Michael B. Ryan, Allison E. Girasole, Andrew J. Flores, Emily L. Twedell, Matthew M. McGregor, Rea Brakaj, Ronald F. Paletzki, Thomas S. Hnasko, Charles R. Gerfen, and Alexandra B. Nelson

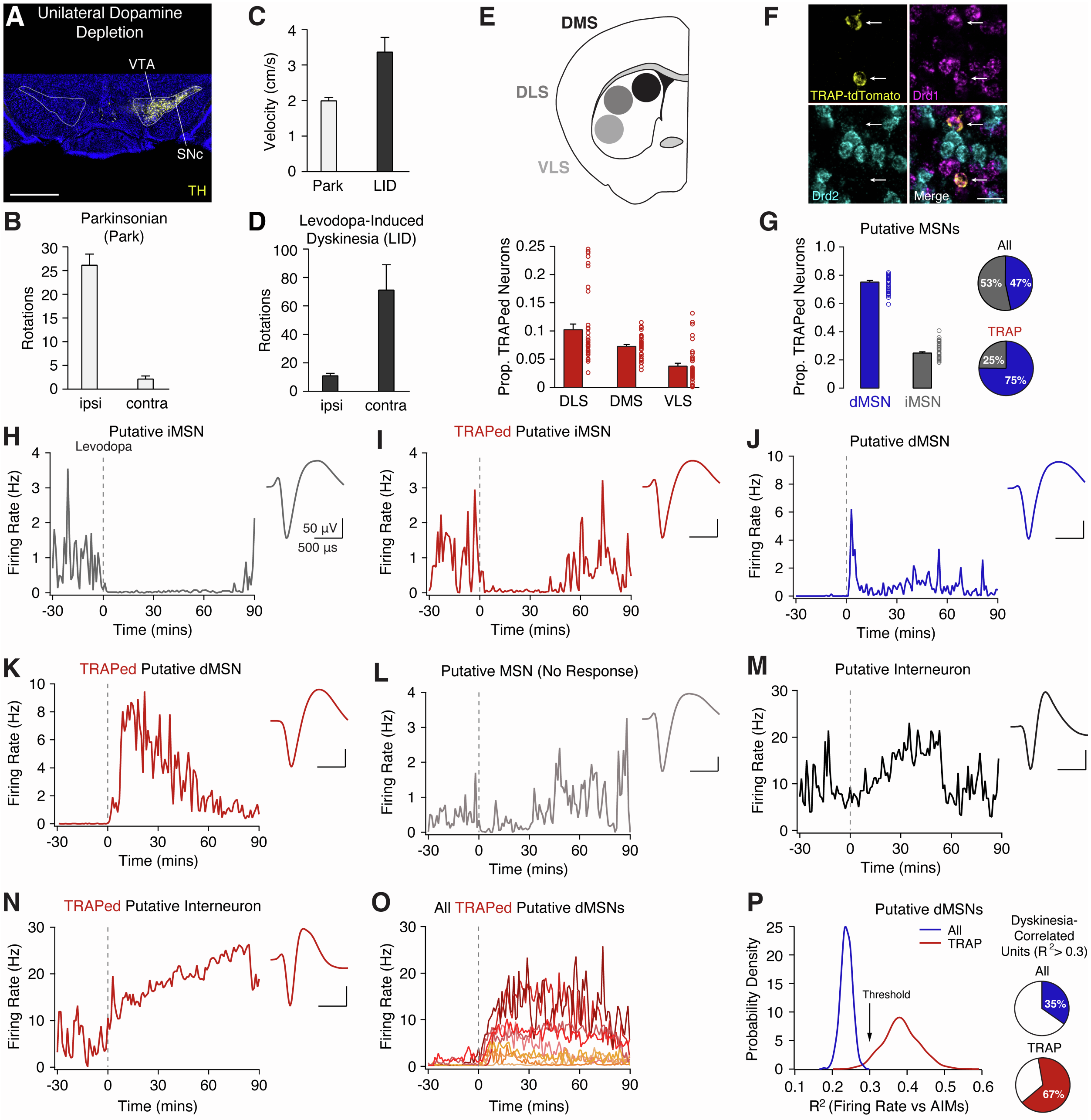

Figure S1: Optogenetically Identified TRAPed Striatal Neurons Show Differential Responses to Levodopa In *Vivo*. Related to Figure 1.

TRAPed striatal single-units were recorded in freely moving parkinsonian mice using an optogenetic labeling approach. **(A)** Representative histological section showing tyrosine-hydroxylase (TH) staining in the midbrain of a hemiparkinsonian mouse. Scale bar = 1 mm. **(B-C)** Ipsilesional (ipsi) and contralesional (contra) rotations over 10 minutes in parkinsonian mice **(B)** before and **(C)** 20 minutes after levodopa. **(D)** Movement velocity of parkinsonian mice before (Park) and 20 minutes after levodopa injection (LID). **(E)** Quantification of TRAPed neuron expression by region: dorsolateral (DLS), dorsomedial (DMS), and ventrolateral (VLS) striatum. Relative proportion of striatal neurons that are TRAPed, identified using *in situ* hybridization for D1 (Drd1a) and D2 (Drd2a) receptor mRNA. N=5, n=39. **(F)** Representative high magnification image of *in situ* hybridization for TRAP (tdTomato), Drd1a (D1R), and Drd2a (D2R) mRNA. Arrows indiciate TRAP+ cells. Scale bar = 25 *μ*m. **(G)** Relative proportion of TRAPed neurons that are putative dMSNs (D1R+) or iMSNs (D2R+) (left). Pie charts comparing proportions of putative dMSNs and iMSNs in TRAPed and unTRAPed striatal neurons (right). N=5, n=39. N=animals. N=slices. **(H-N)** Representative single units from parkinsonian mice treated with levodopa. Left: single unit firing rates, aligned to levodopa administration at t=0. Right:

average waveform. **(H)** Unlabeled and **(I)** optogenetically labeled TRAPed putative iMSNs. **(J)** Unlabeled and **(K)** optogenetically labeled TRAPed putative dMSNs. **(L)** Unlabeled MSN with no response to levodopa **(M)** Unlabeled and **(N)** optogenetically labeled TRAPed putative interneurons. **(O)** Firing rates of all (n=9) TRAPed putative dMSNs aligned to levodopa injection at t=0. **(P)** Left: Probability density plot of average correlation (R^2) obtained from bootstrapping all putative dMSN (blue) and optogenetically labeled TRAPed putative dMSN data (red). Right: Proportion of all (n=170, N=20) and TRAPed (n=9, N=7) putative dMSNs with a significant correlation ($R^2 > 0.3$) between firing rate and dyskinesia score. n=single-units, N=mice. Data presented as mean ± SEM.

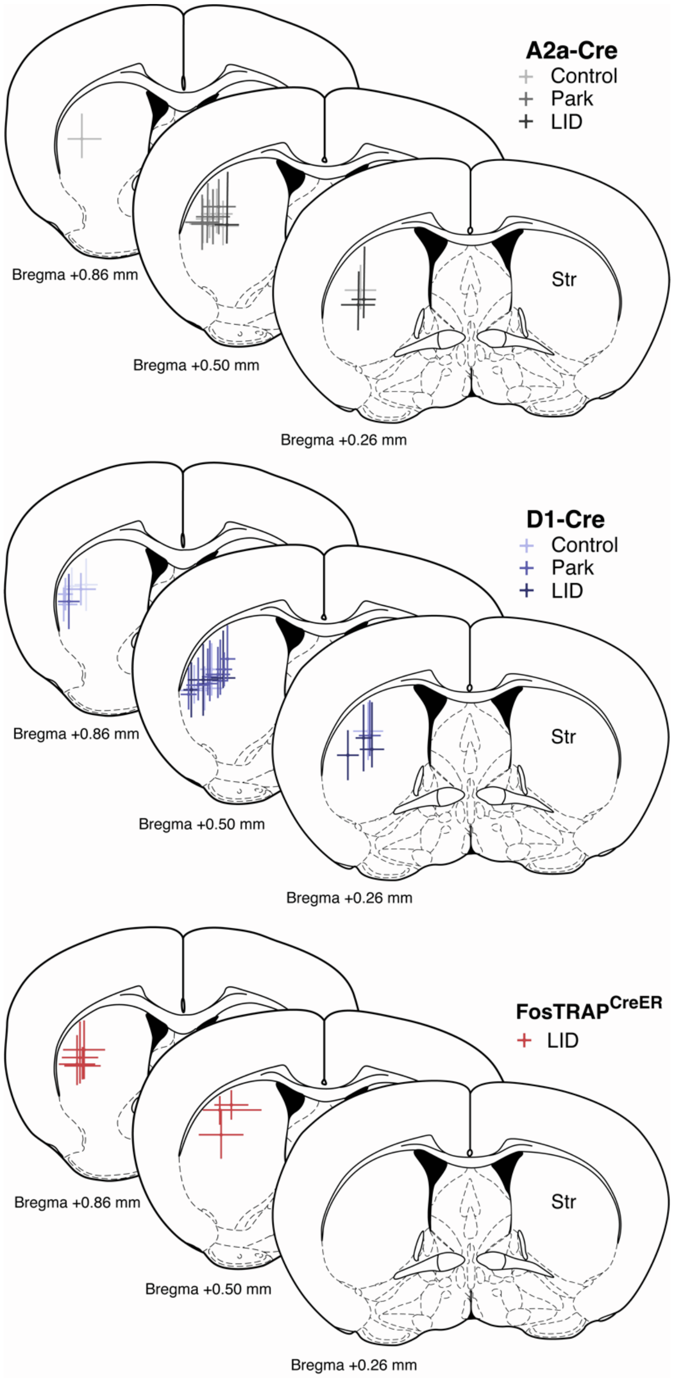

Figure S2. Distribution of Starter Cells in Rabies Injected Brains. Related to Fig 2.
Striatal schematics representing the center and spread of "starter" cells (sTpEpB, green) in rabies injected brains across A2a-Cre (top), D1-Cre (middle), and FosTRAP-CreER (bottom) mice across experimental conditions (Control, Park, and LID). The spread within the striatum was assessed by the extent of green fluorescent cells visible in a low-magnification image (overall quantification of starter cells is shown in Fig S3). The shading of the cross refers to experimental condition (light = Control, medium = Park, dark = LID). The center of the cross represents the center of the starter cell injection site and the spread is represented by the x and y distance from the center of the cross. A2a: Control, N=6, Park, N=4, LID, N=3; D1: Control, N=9, Park, N=10, LID, N=6; TRAP: LID, N=7. N= animals.

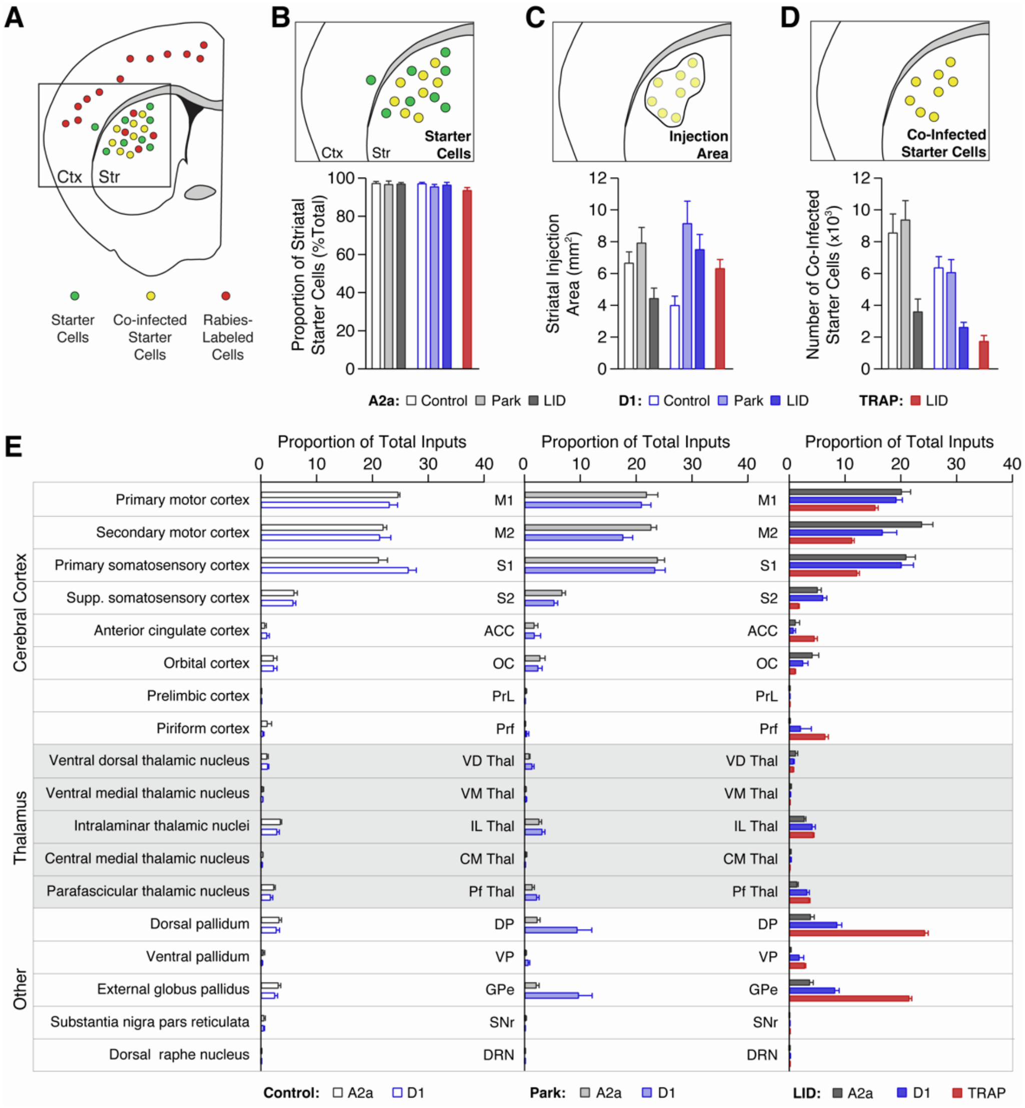

Figure S3. Monosynaptic Rabies Tracing onto Indirect Pathway, Direct Pathway, and TRAPed Striatal Neurons. Related to Fig 2.

A dual viral, Cre-dependent strategy was used to label monosynaptic inputs onto direct pathway, indirect pathway, and TRAPed striatal neurons. **(A)** Top: Coronal schematic of starter (green), rabies-labeled (red), and co-infected starter (yellow) cells. Bottom: Description of the groups and treatment conditions. **(B-D)** Top: coronal schematic showing quantification approach. Bottom: quantification of injection site. **(B)** The proportion of striatal starter cells (sTpEpB positive) compared to all brain-wide starter cells. **(C)** Striatal injection area, quantified by the extent of co-infected cells in the striatum. **(D)** The total number of co-infected striatal neurons. **(E)** The proportion of extra-striatal rabies labeled cell bodies for a given brain region relative to all extra-striatal presynaptic cell bodies brain-wide. A2a: Control, N=6, Park, N=4, LID, N=3; D1: Control, N=9, Park, N=10, LID, N=6; TRAP: LID, N=8. Data presented as mean ± SEM. N= animals.

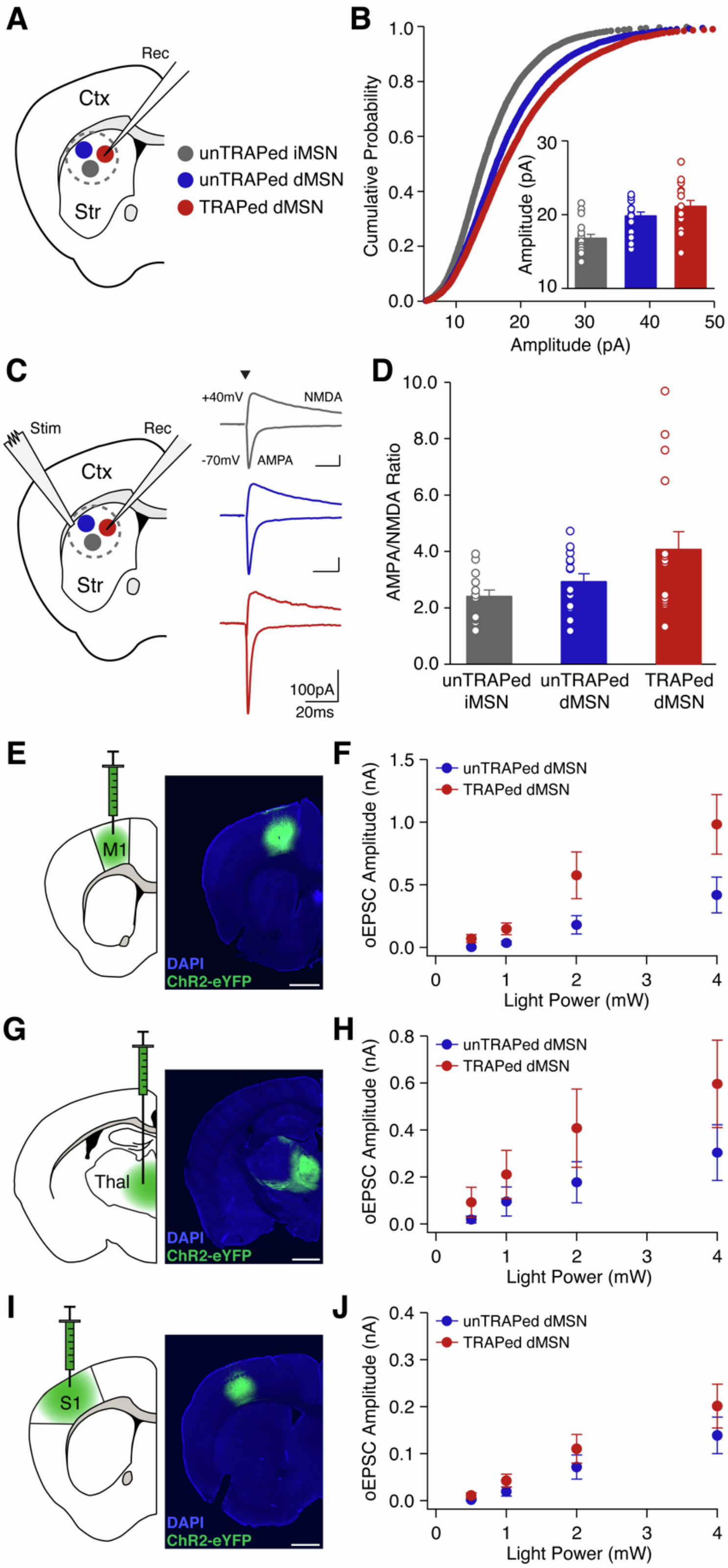
A
Rec
Ctx
Str
unTRAPed iMSN
unTRAPed dMSN
TRAPed dMSN
B
Cumulative Probability
Amplitude (pA)
C
Stim
Rec
Ctx
Str
+40mV
NMDA
-70mV
AMPA
100pA
20ms
D
AMPA/NMDA Ratio
unTRAPed iMSN
unTRAPed dMSN
TRAPed dMSN
E
M1
DAPI
ChR2-eYFP
F
unTRAPed dMSN
TRAPed dMSN
oEPSC Amplitude (nA)
Light Power (mW)
G
Thal
DAPI
ChR2-eYFP
H
unTRAPed dMSN
TRAPed dMSN
oEPSC Amplitude (nA)
Light Power (mW)
I
S1
DAPI
ChR2-eYFP
J
unTRAPed dMSN
TRAPed dMSN
oEPSC Amplitude (nA)
Light Power (mW)

Figure S4. Increased Motor Cortical and Thalamic Excitatory Transmission onto TRAPed dMSNs. Related to Figure 3.

Excitatory inputs to striatal dMSNs were compared in *ex vivo* brain slices from the dorsolateral striatum of FosTRAPxAi14xD2-GFP (FAD) mice. **(A)** UnTRAPed iMSNs (gray), unTRAPed dMSNs (blue), and TRAPed dMSNs (red) were targeted for voltage-clamp recordings of miniature excitatory postsynaptic currents (mEPSCs). **(B)** Cumulative probability of mEPSC amplitudes. Inset: average mEPSC amplitude (unTRAPed iMSNs: n=21, N=8; unTRAPed dMSNs: n=20, N=7; TRAPed dMSNs: n=20, N=8). **(C)** (Left) UnTRAPed iMSNs (gray), unTRAPed dMSNs (blue), and TRAPed dMSNs (red) were targeted for voltage-clamp recordings of electrically evoked EPSCs. (Right) Representative EPSCs recorded at holding potentials of -70mV and +40mV to measure AMPA- and NMDA-mediated currents, respectively. Arrowheads denote timing of the electrical stimulus. **(D)** AMPA/NMDA ratio in unTRAPed iMSNs (n=14, N=7), unTRAPed dMSNs (n=16, N=9), and TRAPed dMSNs (n=16, N=8). N=mice, n=cells. (**E-J)** Excitatory inputs onto sequentially recorded TRAPed and unTRAPed dMSN pairs were compared using an optogenetic approach. **(E,G,I)** Schematic diagrams (left) and histological sections (right) showing expression of Synapsin-ChR2-eYFP in **(E)** primary motor cortex (M1), **(G)** intralaminar thalamus (Thal), and **(I)** primary somatosensory cortex (S1). Scale bar = 1 mm. **(F,H,J)** Average oEPSC amplitude versus light power for unTRAPed (blue) and TRAPed (red) dMSNs following stimulation of inputs from **(F)** M1: n=19, N=4. **(H)** Thal: n=15, N=5. **(J)** S1: n=13, N=7. n=pairs, N=mice. Data presented as mean ± SEM.

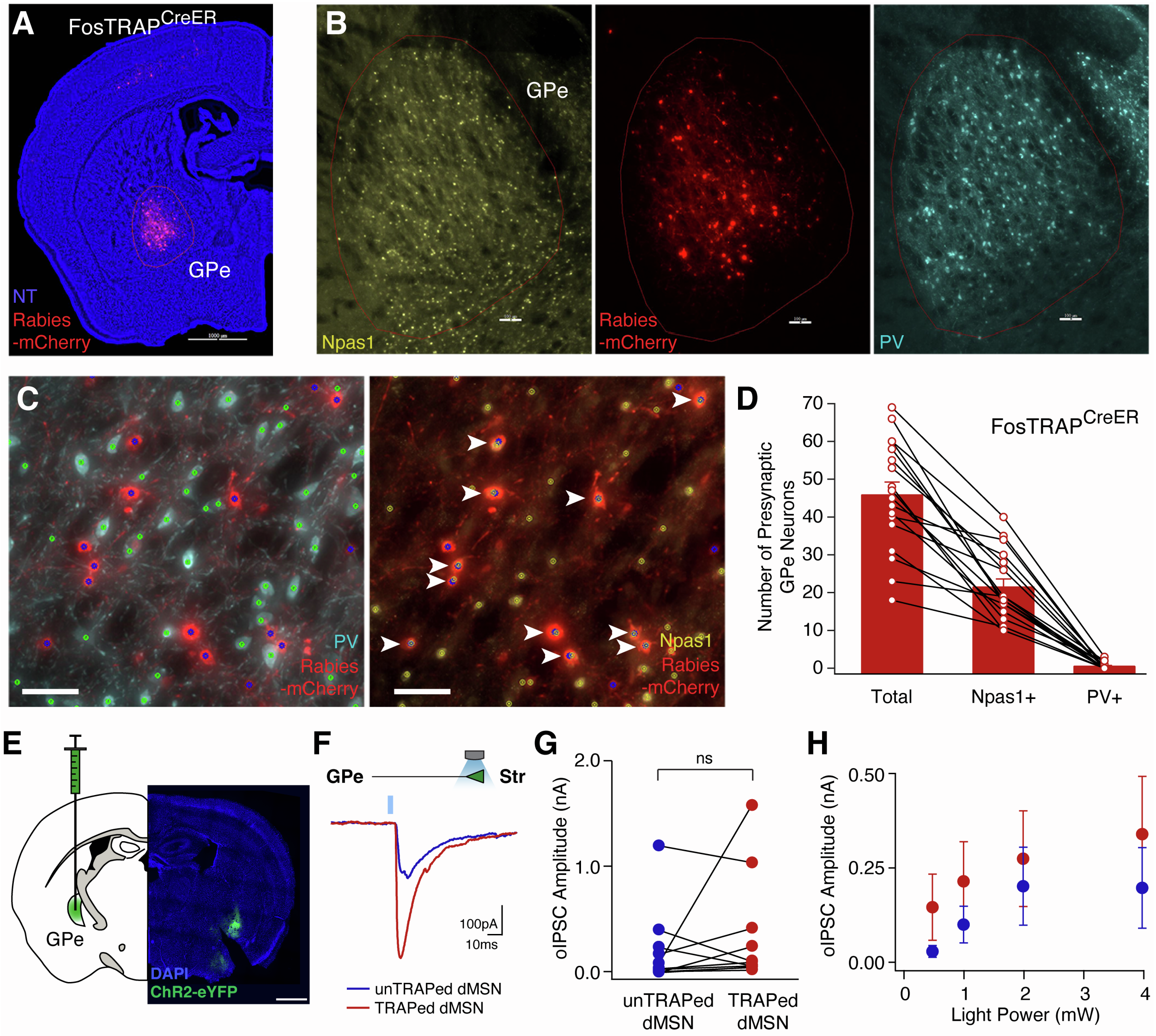

Figure S5. Quantification of GPe Synaptic Input to the Striatum. Related to Figures 2 and 3.
(A-D) Quantification of rabies-labeled presynaptic GPe neurons in Fos-CreER mice. **(A)** Low magnification of coronal section with rabies-labeled cells in red. Scale bar=1mm. **(B)** Higher magnification image of GPe in panel A, showing Npas1 (yellow), rabies-labeled cells (red) and parvalbumin (PV, blue). Scare bar=100 μm. **(C)** High magnification image of GPe showing colocalization between rabies-labeled cells and PV (left) and Npas1 (right). Arrowheads denote co-labeled cells. Scale bar=50 μm **(D)** Quantification of Npas1+ and PV+ rabies-labeled neurons in the GPe of FosTRAP-CreER mice. N=3 mice, n=18 sections. **(E-H)** Inhibitory GPe inputs to striatal dMSNs were compared in *ex vivo* brain slices from the dorsolateral striatum of FosTRAP;Ai14;D2-GFP (FAD) mice. **(E)** Coronal schematic (left) and postmortem histology (right) showing viral expression of ChR2-eYFP in the GPe. Scale bar = 1 mm. **(F)** Representative example of optically-evoked IPSCs (oIPSCs) for sequentially recorded unTRAPed and TRAPed dMSN pairs in the same field of view. **(G)** Average oIPSC amplitude at 4mW for unTRAPed and TRAPed dMSNs. **(H)** Average oIPSC amplitude versus light power for unTRAPed (blue) and TRAPed (red) dMSNs (n=11, N=5). n=cells, N=mice. Data presented as mean ± SEM.

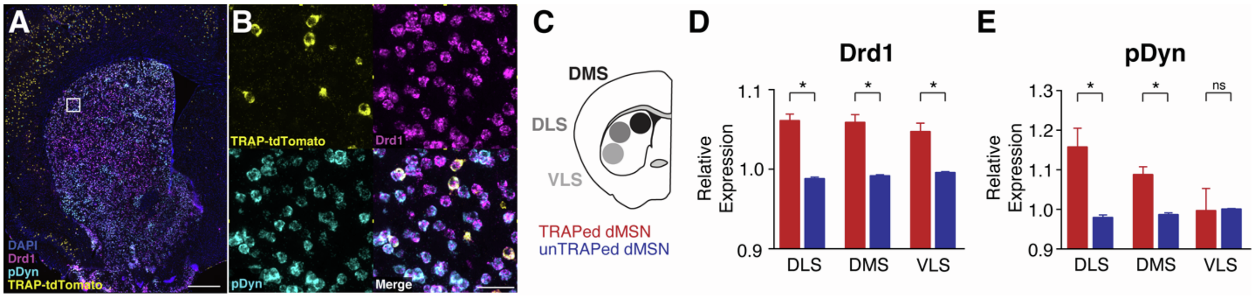

Figure S6. Increased pDyn and D1 Receptor mRNA in TRAPed dMSNs. Related to Figure 4.
Fluorescent *in situ* hybridization to quantify prodynorphin (pDyn) mRNA in levodopa-treated FosTRAP:Ai14 mice. **(A)** Low magnification of coronal section labeled for DAPI, D1R (Drd1), pDyn (prodynorphin), and TRAP (tdTomato) mRNA. Scale bar = 1 mm. **(B)** High magnification of inset shown in (A). Scale bar = 50 μm. **(C)** Schematic of striatal subregion quantification. **(D-E)** Expression of molecular markers for TRAPed and unTRAPed dMSNs, normalized by the average expression of all dMSNs. **(D)** Quantification of relative expression of D1R mRNA in TRAPed and unTRAPed dMSNs across striatal subregions (n=39, N=5). **(E)** Quantification of relative expression of pDyn mRNA in TRAPed and unTRAPed dMSNs across striatal subregions (n=31, N=4). n=slices, N=mice. DLS=dorsolateral striatum, DMS=dorsomedial striatum, VLS=ventrolateral striatum. Data presented as mean ± SEM. *$p<0.05$, Wilcoxon Signed Rank Test, Bonferroni corrected for multiple comparisons.

Key Experiments	Figure	Type of Comparison	Statistical Test	n (units/cells)	N (animals)	Comparison values (± SEM) & p value	Planned Sample Size (from power calculation)
**Baseline firing rate of MSNs in Park state, in vivo**	1k	Between-group	MWU	9 (opto labeled TRAP dMSN), 170 (dMSN)	7 (TRAP), 19 (dMSN)	TRAP dMSNs: 0.31 ± 0.16 Hz; All dMSNs: 0.24 ± 0.03 Hz; p=0.903	n = 10 cells
**Change in firing rate of MSNs in LID state, in vivo**	1k	Between-group	MWU	9 (opto labeled TRAP dMSN), 170 (dMSN)	7 (TRAP), 19 (dMSN)	TRAP dMSNs: 6.09 ± 1.52 Hz, All dMSNs: 2.61 ± 0.25 Hz; p=0.017	n = 10 cells
**Correlation of MSN firing rate to dyskinesia severity**	1o, S1j	Between-group	MWU	9 (opto labeled TRAP dMSN), 170 (dMSN)	7 (TRAP), 19 (dMSN)	TRAP dMSNs: R2 = 0.38 ± 0.05; All dMSNs: R2 = 0.24 ± 0.02; p=0.021	n = 10 cells
**Evoked firing rate of MSNs in Park state, ex vivo**	4e, Table S2	Between-group	rmANOVA	22 (TRAP dMSN), 17 (un T dMSN), 17 (unT iMSN)	14 (TRAP dMSN), 13 (un T dMSN), 11 (unT iMSN)	F(10,230)=2.598, p=0.005, unT dMSN vs. unT iMSN: p=0.197, TRAP dMSN vs. unT dMSN: p=0.999, TRAP dMSN vs unT iMSN: p=0.187	n=15 cells/group
**Changes in evoked firing rate of MSNs after SKF-81297, ex vivo**	4f-g, Table S3	Within-cell	rmANOVA	14 (TRAP dMSN), 11 (un T dMSN), 9 (unT iMSN)	10 (TRAP dMSN), 9 (un T dMSN), 6 (unT iMSN)	unT iMSN: F(5,80)=0.362, p=0.874, unT dMSN: F(5,110)=0.370, p=0.839, TRAP dMSN: F(5,130)=2.375, p=0.0425	n=10 cells/group
**Total number of presynaptic inputs onto MSNs (control)**	2h	Between-group	MWU	n/a	6 (A2a), 9 (D1)	Control: iMSN vs dMSN, p=0.114	N = 5 mice/group
**Number of cortical inputs onto iMSNs (all groups)**	2i	Between-group	KW	n/a	Control: 6 (A2a), Park: 4 (A2a), LID: 3 (A2a)	χ2(2) 8.881, p=0.012	N = 5 mice/group
**Number of cortical inputs onto iMSNs (control vs. Park)**	2i	Between-group	HSD	n/a	Control: 6 (A2a), Park: 4 (A2a)	Control vs Park, p=0.0498	N = 5 mice/group
**Number of cortical inputs onto iMSNs (Park vs. LID)**	2i	Between-group	HSD	n/a	Park: 4 (A2a), LID: 3 (A2a)	Park vs LID, p=0.970	N = 5 mice/group
**Number of cortical inputs onto iMSNs (control vs. LID)**	2i	Between-group	HSD	n/a	Park: 4 (A2a), LID: 3 (A2a)	Healthy vs LID, p=0.024	N = 5 mice/group
**Number of thalamic inputs onto iMSNs (all groups)**	2j	Between-group	KW	n/a	Control: 6 (A2a), Park: 4 (A2a), LID: 3 (A2a)	χ2(2) 8.214, p=0.017	N = 5 mice/group
**Number of thalamic inputs onto iMSNs (control vs. Park)**	2j	Between-group	HSD	n/a	Control: 6 (A2a), Park: 4 (A2a)	Control vs Park, p=0.184	N = 5 mice/group
**Number of thalamic inputs onto iMSNs (Park vs. LID)**	2j	Between-group	HSD	n/a	Park: 4 (A2a), LID: 3 (A2a)	Park vs LID, p=0.343	N = 5 mice/group
**Number of thalamic inputs onto iMSNs (control vs. LID)**	2j	Between-group	HSD	n/a	Control: 6 (A2a), LID: 3 (A2a)	Control vs LID, p=0.0151	N = 5 mice/group
**Number of GPe inputs onto iMSNs (all groups)**	2k	Between-group	KW	n/a	Control: 6 (A2a), Park: 4 (A2a), LID: 3 (A2a)	χ2(2) 8.2571, p=0.016	N = 5 mice/group
**Number of GPe inputs onto iMSNs (control vs. Park)**	2k	Between-group	HSD	n/a	Control: 6 (A2a), Park: 4 (A2a)	Control vs Park, p=0.1714	N = 5 mice/group
**Number of GPe inputs onto iMSNs (Park vs. LID)**	2k	Between-group	HSD	n/a	Park: 4 (A2a), LID: 3 (A2a)	Park vs LID, p=0.0286	N = 5 mice/group
**Number of GPe inputs onto iMSNs (control vs. LID)**	2k	Between-group	HSD	n/a	Control: 6 (A2a), LID: 3 (A2a)	Control vs LID, p=0.019	N = 5 mice/group
**Number of cortical inputs onto dMSNs (all groups)**	2i	Between-group	KW	n/a	Control: 9 (D1), Park: 10 (D1), LID: 6 (D1)	χ2(2) 4.185, p=0.123	N = 5 mice/group
**Number of thalamic inputs onto dMSNs (all groups)**	2j	Between-group	KW	n/a	Control: 9 (D1), Park: 10 (D1), LID: 6 (D1)	χ2(2) 1.161, p=0.560	N = 5 mice/group
**Number of Gpe inputs onto dMSNs (all groups)**	2k	Between-group	KW	n/a	Control: 9 (D1), Park: 10 (D1), LID: 6 (D1)	χ2(2) 12.309, p=0.002	N = 5 mice/group
**Number of GPe inputs onto dMSNs (control vs. Park)**	2k	Between-group	HSD	n/a	Control: 9 (D1), Park: 10 (D1)	Control vs Park, p=0.0497	N = 5 mice/group

**Number of GPe inputs onto dMSNs (Park vs. LID)**	2k	Between-group	HSD	n/a	Park: 10 (D1), LID: 6 (D1)	Park vs LID, p=0.353	N = 5 mice/group
**Number of GPe inputs onto dMSNs (control vs. LID)**	2k	Between-group	HSD	n/a	Control: 9 (D1) , LID: 6 (D1)	Control vs LID, p<0.001	N = 5 mice/group
**Total number of presynaptic inputs on dMSN (TRAP vs. LID)**	2h	Between-group	MWU	n/a	LID: 6 (D1), LID: 6 (TRAP)	TRAP vs dMSN, p=0.132	N = 5 mice/group
**Number of cortical inputs onto dMSNs (TRAP vs. LID)**	2i	Between-group	MWU	n/a	LID: 6 (D1), LID: 6 (TRAP)	TRAP vs dMSN, p=0.009	N = 5 mice/group
**Number of thalamic inputs onto dMSNs (TRAP vs. LID)**	2j	Between-group	MWU	n/a	LID: 6 (D1), LID: 6 (TRAP)	TRAP vs dMSN, p=0.041	N = 5 mice/group
**Number of GPe inputs onto dMSNs (TRAP vs. LID)**	2k	Between-group	MWU	n/a	LID: 6 (D1), LID: 6 (TRAP)	TRAP vs dMSN, p=0.240	N = 5 mice/group
**mEPSC amplitudes of unTRAP dMSNs vs unTRAPed iMSNs**	S4b	Between-group	MWU	20 (unT dMSN), 21 (unT iMSN)	7 (un T dMSN), 8 (unT iMSN)	unT dMSN: 19.89 ± 0.47 pA, unT iMSN: 16.86 ± 0.54 pA, p<0.001	n = 20 cells/group
**mEPSC amplitudes of unTRAP dMSNs vs TRAPed dMSNs**	S4b	Between-group	MWU	20 (TRAP dMSN), 20 (unT dMSN)	8 (TRAP dMSN), 7 (unT dMSN)	TRAP dMSN: 21.22 ± 0.67 pA, unT dMSN: 19.89 ± 0.47 pA, p=0.102	n = 20 cells/group
**mEPSC frequency of unTRAP dMSNs vs unTRAPed iMSNs**	3b	Between-group	MWU	20 (unT dMSN), 21 (unT iMSN)	7 (unT dMSN), 8 (unT iMSN)	unT iMSN: 2.80 ± 0.36 Hz, unT dMSN: 3.87 ± 0.41 Hz, p=0.034	n = 20 cells/group
**mEPSC frequency of unTRAP dMSNs vs TRAPed dMSNs**	3b	Between-group	MWU	20 (TRAP dMSN), 20 (unT dMSN)	8 (TRAP dMSN), 7 (unT dMSN)	TRAP dMSN: 5.41 ± 0.47 Hz, unT dMSN: 3.87 ± 0.41 Hz, p=0.021	n = 20 cells/group
**Evoked EPSCs, AMPA/NMDA ratio in dMSNs**	S4d	Between-group	MWU	16 (TRAP dMSN), 16 (un T dMSN), 14 (unT iMSN)	8 (TRAP dMSN), 9 (unT dMSN), 7 (unT iMSN)	TRAP dMSN: 4.07 ± 0.63, unT dMSN: 2.92 ± 0.28, p=0.376, RS	n=15 cells/group
**Evoked EPSCs, PPR**	3d	Between-group	rmANOVA, HSD	22 (TRAP dMSN), 18 (un T dMSN), 17 (unT iMSN)	9 (TRAP dMSN), 9 (unT dMSN), 8 (unT iMSN)	F(10,270)=2.493, p=0.007, rmANOVA; unT dMSN vs unT iMSN, p=0.367, unT dMSN vs T dMSN, p=0.0186, unT iMSN vs T dMSN, p<0.001, Tukey	n=15 cells/group
**Optically evoked EPSCs, M1**	3j	Between-group (sequential pairs)	WSR	13	4	T dMSN: 0.98 ± 0.24 nA, unT dMSN: 0.42 ± 0.14 nA, p=0.002	n=15 pairs
**Optically evoked EPSCs, S1**	3p	Between-group (sequential pairs)	WSR	15	7	T dMSN: 0.21 ± 0.05 nA, unT dMSN: 0.13 ± 0.04 nA, p=0.073	n=15 pairs
**Optically evoked EPSCs, thalamus**	3m	Between-group (sequential pairs)	WSR	18	5	T dMSN: 0.60 ± 0.18 nA, unT dMSN: 0.31 ± 0.12 nA, p=0.008	n=15 pairs
**Optically evoked IPSCs, Gpe**	S5g	Between-group (sequential pairs)	WSR	11	5	T dMSN: 0.34 ± 0.15 nA, unT dMSN: 0.20 ± 0.11 nA, p=0.037	n=15 pairs
**In situ hybridization, Drd1a expression unTRAP vs TRAPed dMSNs**	4l	Between-group	WSR	39 (slices)	5	T dMSN: 1.07 ± 0.008 au, unT dMSN: 0.99 ± 0.001 au, p<0.001	N = 5 mice
**In situ hybridization, Drd2a expression unTRAP vs TRAPed dMSNs**	4m	Between-group	WSR	39 (slices)	5	T dMSN: 1.02 ± 0.013 au, unT dMSN: 1.00 ± 0.002 au, p=0.219	N = 5 mice
**In situ hybridization, pDyn expression unTRAP vs TRAPed dMSNs**	4n	Between-group	WSR	31 (slices)	4	T dMSN: 1.17 ± 0.048 au, unT dMSN: 0.98 ± 0.006 au, p<0.001	N = 5 mice
**In situ hybridization, Drd1a expression unTRAP vs TRAPed dMSNs across striatal subregions**	S6d	Between-group	WSR	39 (slices)	5	TdMSN (DLS: 1.06 ± 0.008; DMS: 1.06 ± 0.010; VLS: 1.05 ± 0.010), unT dMSN (DLS: 0.99 ± 0.002; DMS: 0.99 ± 0.001; VLS: 1.00 ± 0.001), DLS T vs unT dMSN p*<0.001; DMS T vs unT dMSN p*<0.001; VLS T vs unT dMSN p*<0.001	N = 5 mice
**In situ hybridization, pDyn expression unTRAP vs TRAPed dMSNs across striatal subregions**	S6e	Between-group	WSR	31 (slices)	4	TdMSN (DLS: 1.16 ± 0.047; DMS: 1.09 ± 0.020; VLS: 1.00 ± 0.056), unT dMSN (DLS: 0.98 ± 0.006; DMS: 0.99 ± 0.004; VLS: 1.00 ± 0.001), DLS T vs unT dMSN p*<0.001; DMS T vs unT dMSN p*<0.001; VLS T vs unT dMSN p*=1.75	N = 5 mice

Table S1. Statistical Table. Each row in the table provides key information regarding a statistical comparison made in the manuscript, including the Figure, statistical test, n/N, p value, and planned sample size based on power calculation. Abbreviations: WSR (Wilcoxon Signed Rank Test); MWU (Mann-Whitney U Test); rmANOVA (repeated measures analysis of variance); KW (Kruskal-Wallis Test), HSD (Tukey's HSD). *p value corrected for multiple comparisons.

	unTRAPed iMSNs	unTRAPed dMSNs	TRAPed dMSNs	p-value
Input Resistance (MOhm)	79.1 ± 9.5	81.1 ± 5.8	82.3 ± 7.1	0.503
Resting Membrane Potential (mV)	-88.3 ± 1.0	-89.2 ± 0.9	-89.4 ± 0.8	0.542
Action Potential Threshold (mV)	-39.8 ± 2.7	-38.9 ± 1.5	-38.9 ± 1.8	0.834
AHP Amplitude (mV)	15.6 ± 1.1	13.8 ± 1.1	14.7 ± 1.0	0.238
Spike Width (ms)	1.8 ± 0.1	2.0 ± 0.1	2.1 ± 0.1	0.159
Rheobase (pA)	406.1 ± 39.6	341.2 ± 25.1	384.2 ± 7.1	0.447

Table S2. Baseline Passive and Active Membrane Properties of MSNs. Related to Figure 4.
Passive (input resistance and resting membrane potential) and active properties (action potential threshold, after-hyperpolarization amplitude, spike width, and rheobase) of excitability in the absence of dopamine receptor stimulation. Data presented as mean ± SEM.

	unTRAPed iMSNs			unTRAPed dMSNs			TRAPed dMSNs		
	Control	SKF-81297	p-value	Control	SKF-81297	p-value	Control	SKF-81297	p-value
Input Resistance (MOhm)	79.8 ± 13.8	91.8 ± 14.5	0.383	82.0 ± 7.8	99.9 ± 13.4	0.032	89.4 ± 8.7	90.4 ± 8.3	0.925
Resting Membrane Potential (mV)	-89.8 ± 0.8	-86.8 ± 3.1	0.469	-90.5 ± 1.1	-89.0 ± 1.3	0.320	-90.3 ± 1.0	-90.0 ± 1.6	0.791
Action Potential Threshold (mV)	-39.5 ± 3.5	-38.5 ± 3.0	0.313	-37.5 ± 1.9	-35.9 ± 1.6	0.910	-38.9 ± 2.3	-38.7 ± 2.8	0.910
AHP Amplitude (mV)	21.4 ± 3.2	20.8 ± 3.3	0.148	28.8 ± 3.3	22.8 ± 2.5	0.700	27.1 ± 2.3	22.9 ± 3.9	0.204
Spike Width (ms)	1.8 ± 0.2	2.2 ± 0.7	0.313	2.0 ± 0.1	2.1 ± 0.1	0.049	2.1 ± 0.6	2.2 ± 0.7	0.233
Rheobase (pA)	440.0 ± 51.5	405 ± 51.9	0.250	345.8 ± 29.2	312.5 ± 28.3	0.125	**371.4 ± 29.0**	**321.4 ± 31.8**	**0.0078**

Table S3. Passive and Active Membrane Properties of MSNs in Response to the D1-receptor specific agonist, SKF-81297. Related to Figure 4.
Passive (input resistance and resting membrane potential) and active properties (action potential threshold, after-hyperpolarization amplitude, spike width, and rheobase) of excitability before (Control) and 10-15 minutes after bath application of a D1-specific agonist (SKF-81297). Bolded values represent significant p-values, following Bonferroni correction for multiple comparisons. Data presented as mean ± SEM.
